# Measurement of H_2_O_2_ within Living *Drosophila* during Aging Using a Ratiometric Mass Spectrometry Probe Targeted to the Mitochondrial Matrix

**DOI:** 10.1016/j.cmet.2011.02.003

**Published:** 2011-03-02

**Authors:** Helena M. Cochemé, Caroline Quin, Stephen J. McQuaker, Filipe Cabreiro, Angela Logan, Tracy A. Prime, Irina Abakumova, Jigna V. Patel, Ian M. Fearnley, Andrew M. James, Carolyn M. Porteous, Robin A.J. Smith, Saima Saeed, Jane E. Carré, Mervyn Singer, David Gems, Richard C. Hartley, Linda Partridge, Michael P. Murphy

**Affiliations:** 1 Medical Research Council Mitochondrial Biology Unit, Hills Road, Cambridge CB2 0XY, UK; 2 Department of Genetics, Evolution, and Environment, Institute of Healthy Ageing, University College London, Gower Street, London WC1E 6BT, UK; 3 Department of Medicine, Bloomsbury Institute of Intensive Care Medicine, University College London, Gower Street, London WC1E 6BT, UK; 4 Centre for the Chemical Research of Ageing, WestCHEM School of Chemistry, University of Glasgow, Glasgow, G12 8QQ, UK; 5 Department of Chemistry, University of Otago, P.O. Box 56, Dunedin 9054, New Zealand; 6 Department of Biochemistry, University of Otago, P.O. Box 56, Dunedin 9054, New Zealand

## Abstract

Hydrogen peroxide (H_2_O_2_) is central to mitochondrial oxidative damage and redox signaling, but its roles are poorly understood due to the difficulty of measuring mitochondrial H_2_O_2_ in vivo. Here we report a ratiometric mass spectrometry probe approach to assess mitochondrial matrix H_2_O_2_ levels in vivo. The probe, MitoB, comprises a triphenylphosphonium (TPP) cation driving its accumulation within mitochondria, conjugated to an arylboronic acid that reacts with H_2_O_2_ to form a phenol, MitoP. Quantifying the MitoP/MitoB ratio by liquid chromatography-tandem mass spectrometry enabled measurement of a weighted average of mitochondrial H_2_O_2_ that predominantly reports on thoracic muscle mitochondria within living flies. There was an increase in mitochondrial H_2_O_2_ with age in flies, which was not coordinately altered by interventions that modulated life span. Our findings provide approaches to investigate mitochondrial ROS in vivo and suggest that while an increase in overall mitochondrial H_2_O_2_ correlates with aging, it may not be causative.

## Highlights

► A mitochondria-targeted mass spectrometry probe measures mitochondrial H_2_O_2_ in vivo ► Overall mitochondrial H_2_O_2_ increases with age but can be independent of life span ► Increased physical activity leads to a decrease in mitochondrial H_2_O_2_ ► Hypotheses dependent on overall mitochondrial ROS can now be assessed in vivo

## Introduction

Generation of the reactive oxygen species (ROS) H_2_O_2_ within the mitochondrial matrix is central to pathological oxidative damage and redox signaling, yet little is known about the extent or regulation of mitochondrial ROS levels in vivo (Balaban et al., 2005, Murphy, 2009). Mitochondrial ROS are generally assessed using fluorescent probes (Belousov et al., 2006, Dickinson and Chang, 2008, Rhee et al., 2010), but these are only applicable to optically accessible systems. Consequently ROS changes in vivo are usually inferred indirectly from oxidative damage markers (Beckman and Ames, 1998), but this is questionable because damage alters in response to repair and turnover pathways (Murphy, 2009). Furthermore, many signaling effects of ROS in vivo are due to their concentration and are independent of damage. Therefore, measurements of mitochondrial ROS levels within living organisms are essential. To address this challenge, we have developed a mitochondria-targeted mass spectrometry probe approach.

**Figure 1:**
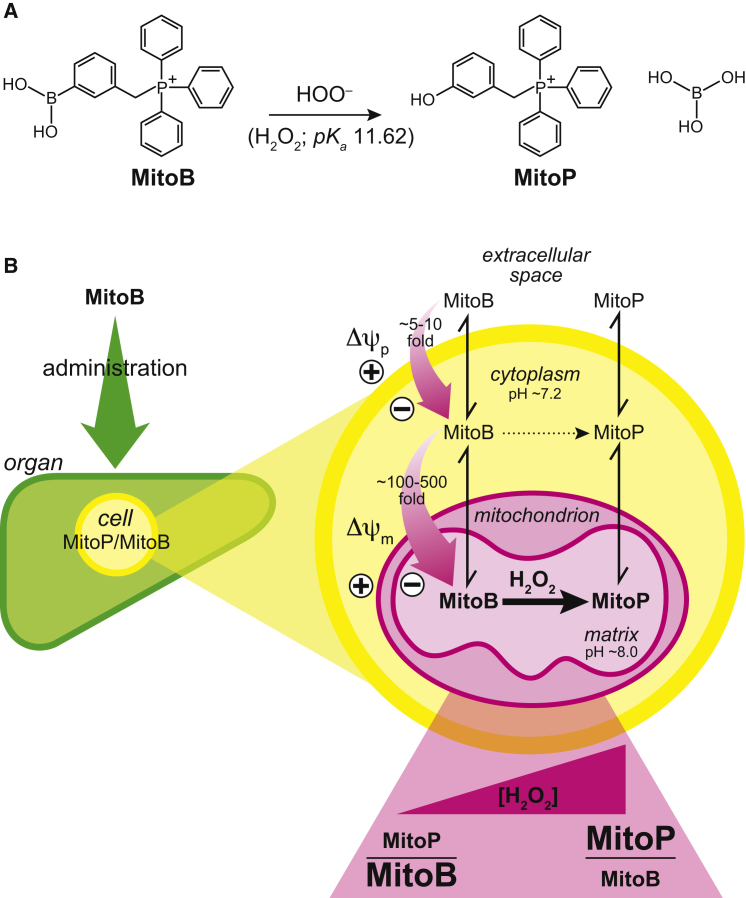
Rationale for the Development of a Mitochondrial H_2_O_2_ Probe (A) Structure of mitochondria-targeted boronic acid, MitoB, and its phenol product, MitoP, formed by reaction with HOO^-^ (the conjugate base of H_2_O_2_). (B) MitoB uptake into mitochondria within tissues. MitoB is first taken up into cells driven by the plasma membrane potential (*Δψ_p_*), and then accumulates inside the mitochondrial matrix driven by the mitochondrial membrane potential (*Δψ_m_*), resulting in a several-hundred-fold accumulation, as predicted by the Nernst equation. Within mitochondria, MitoB is converted to MitoP in response to H_2_O_2_. The reaction with H_2_O_2_ is further enhanced by the higher pH of the matrix relative to the cytoplasm, thus the rate of MitoB to MitoP conversion is an indicator of the mitochondrial H_2_O_2_ concentration. To measure the MitoP/MitoB ratio, the tissue is spiked with deuterated ISs, extracted, and then quantified by LC-MS/MS.

The strategy (Figure 1) is based on the ability of the lipophilic triphenylphosphonium (TPP) cation to pass rapidly through biological membranes and accumulate several-hundred-fold within mitochondria in vivo, driven by the membrane potential (*Δψ_m_*) (Murphy and Smith, 2007). Bioactive functionalities covalently attached to TPP are thereby selectively delivered to mitochondria in vivo (Murphy and Smith, 2007). To make a mitochondria-targeted ROS probe, we conjugated TPP to an arylboronic acid to generate MitoBoronic acid (MitoB; Figure 1A). The arylboronic acid reactive component was chosen based on the pioneering work of Chang (Dickinson and Chang, 2008, Miller et al., 2007), who showed that this moiety reacts selectively with H_2_O_2_ within cells to form a phenol, thereby making H_2_O_2_-specific fluorescent probes. A further advantage of arylboronic acid is that the active oxidant is the conjugate base of H_2_O_2_ (*pK_a_* = 11.62 at 25°C). Consequently the reaction of MitoB with H_2_O_2_ should be faster in the mitochondrial matrix (pH ∼8.0) compared to the cytosol (pH ∼7.2), further enhancing its specificity for mitochondrial H_2_O_2_. Peroxynitrite (ONOO^-^) rapidly converts arylboronates to phenols (Sikora et al., 2009), so MitoB will also respond to mitochondrial ONOO^-^, which may occur when superoxide and nitric oxide (NO) are present together. Thus the extent of MitoB conversion to its phenol product, MitoP, in vivo will reflect the mitochondrial matrix H_2_O_2_ and ONOO^-^ concentrations.

A crucial aspect of this approach is the use of mass spectrometry to measure the MitoP/MitoB ratio. This allows application to whole organisms by chemical extraction, rather than being limited to cells or tissue surfaces, as with optical approaches. To ensure accurate quantification, it is essential to include deuterated internal standards (ISs) of MitoB and MitoP to correct for variability during extraction and detection. An important additional advantage of using TPP is that its inherent positive charge facilitates sensitive detection by mass spectrometry. Indeed, derivatization with TPP is used to enhance detection sensitivity in mass spectrometry (Woo et al., 2009). Finally, TPP compounds that undergo intramitochondrial reactions rapidly equilibrate with the external medium (Ross et al., 2008). Therefore measurement of the MitoP/MitoB ratio in the extracellular medium may enable minimally invasive measurement of mitochondrial ROS production. Here we show that this approach can be used to assess average mitochondrial matrix H_2_O_2_ concentration in vivo within flies. We then apply this methodology to demonstrate that although average mitochondrial matrix H_2_O_2_ does increase in flies during aging, interventions such as dietary restriction (DR) increase life span without altering mitochondrial H_2_O_2_. These findings have significant implications for how ROS may contribute to aging.

## Results

### Characterization of the MitoB Mass Spectrometry Probe

**Figure 2:**
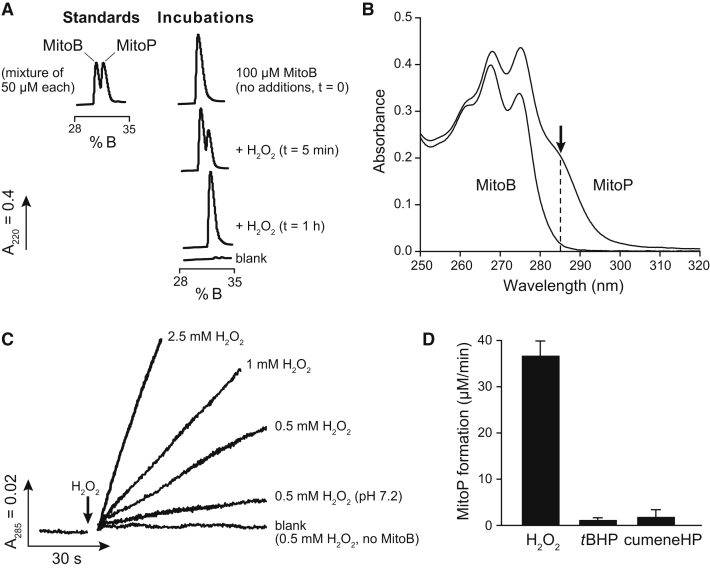
Reaction of MitoB with H_2_O_2_ to Form MitoP (A) Oxidation of MitoB to MitoP assessed by RP-HPLC. MitoB (100 μM) was incubated at 37°C in KCl medium (pH 8.0) with no additions or with 100 μM H_2_O_2_ and then analyzed by RP-HPLC. A mixture of MitoB and MitoP standards (50 μM each) was also analyzed. (B) Absorbance spectra of MitoB and MitoP (100 μM) in KCl medium, showing a large difference in absorption at 285 nm due to the phenol moiety. (C) Progress curves for the reaction of MitoB with H_2_O_2_. The conversion of MitoB (100 μM) to MitoP by reaction with H_2_O_2_ is measured at 285 nm in KCl medium at 37°C (pH 8.0, unless otherwise indicated). (D) Reaction of MitoB with various peroxides. The conversion rate of MitoB (100 μM) to MitoP by reaction with 500 μM of either H_2_O_2_, *tert*-butylhydroperoxide (*t*BHP) or cumene hydroperoxide (cumeneHP) was measured at 285 nm in KCl medium at 37°C, pH 8.0. Data are means ± SD, n = 3.

In vitro reaction of MitoB with H_2_O_2_ gave a single product, identified by reverse phase (RP)-HPLC (Figure 2A) and mass spectrometry as MitoP (*m/z* = 369.1). Addition of excess H_2_O_2_ to MitoB gave a UV absorbance spectrum identical to that of MitoP (Figure 2B). The conversion of MitoB to MitoP by H_2_O_2_ was monitored using the difference in absorbance at 285 nm (Figure 2B), giving a second-order rate constant of ∼9 M^−1^s^−1^ at 37°C and ∼3.8 M^−1^s^−1^ at 25°C, pH 8.0. The reaction is far slower than that of the dominant mitochondrial peroxidase, peroxiredoxin III (k ∼2 × 10^7^ M^−1^s^−1^ [Cox et al., 2010]); therefore, MitoB will not affect physiological levels of H_2_O_2_. The reaction of MitoB with H_2_O_2_ was ∼4-fold faster at the pH of the mitochondrial matrix (8.0) compared to that of the cytosol (7.2) (Figure 2C), consistent with MitoB detecting the conjugate base of H_2_O_2_ and thereby reacting preferentially with mitochondrial H_2_O_2_.

Oxidation of MitoB to MitoP was selective for H_2_O_2_ over simple alkylperoxides (Figure 2D), and it did not react with linoleic acid peroxide (see Figure S2A available online). MitoB was not oxidized to MitoP by superoxide (Figures S2B and S2C), by the NO donor DETA-NONOate (Figure S2D), or by peroxidases (Figures S2E and S2F). Addition of ONOO^-^ (100-250 μM) to MitoB (100 μM) led to the rapid formation of MitoP as assessed by UV spectrophotometry and RP-HPLC (data not shown), consistent with the fast (k ∼10^6^ M^−1^s^−1^) reaction of phenylboronates with ONOO^-^ (Sikora et al., 2009). Therefore, the conversion of MitoB to MitoP is selective for H_2_O_2_ over other biological ROS but will also respond to ONOO^-^.

**Figure 3:**
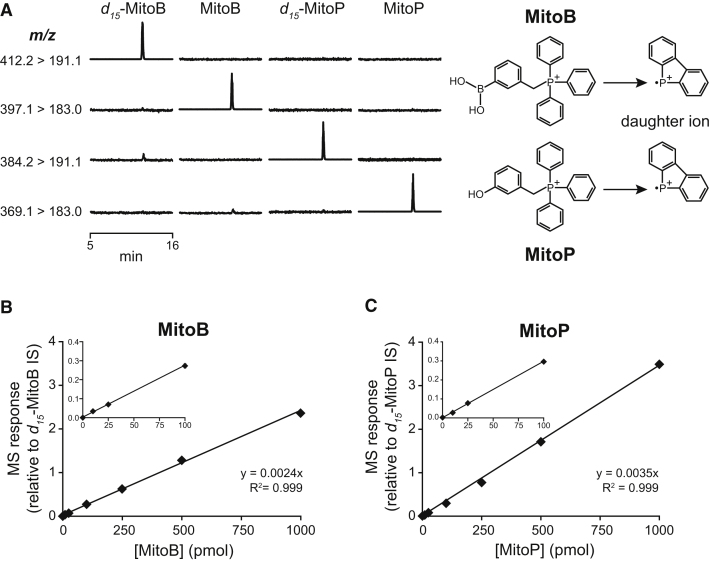
LC-MS/MS Analysis of MitoB and MitoP (A) Typical chromatograms showing the *m/z* transitions measured simultaneously for 20 pmol standards of *d_15_*-MitoB, MitoB, *d_15_*-MitoP, or MitoP. Each trace is normalized to the highest total ion count peak within that trace, hence baseline noise is magnified in background relative to sample traces. (B and C) Example standard curves for MitoB and MitoP detection by LC-MS/MS. In order to quantify the LC-MS/MS data, standard curves were prepared for each experiment by spiking the appropriate biological material with a range of MitoB or MitoP concentrations and the deuterated ISs, and extracting in parallel with the samples. The results presented are from C2C12 cell pellets. Each point is the mean ± range of duplicate measurements.

Both MitoB (Figure S3A) and MitoP (Figure S3C) fragmented during tandem mass spectrometry by pathways expected for alkylTPP cations (Denekamp et al., 1999). This enabled us to establish a sensitive liquid chromatography-tandem mass spectrometry (LC-MS/MS) assay to measure pmol levels of MitoB and MitoP in tissue homogenates relative to deuterated ISs (Figure 3).

### MitoB Accumulates within Mitochondria and Cells, and Responds to H_2_O_2_

**Figure 4:**
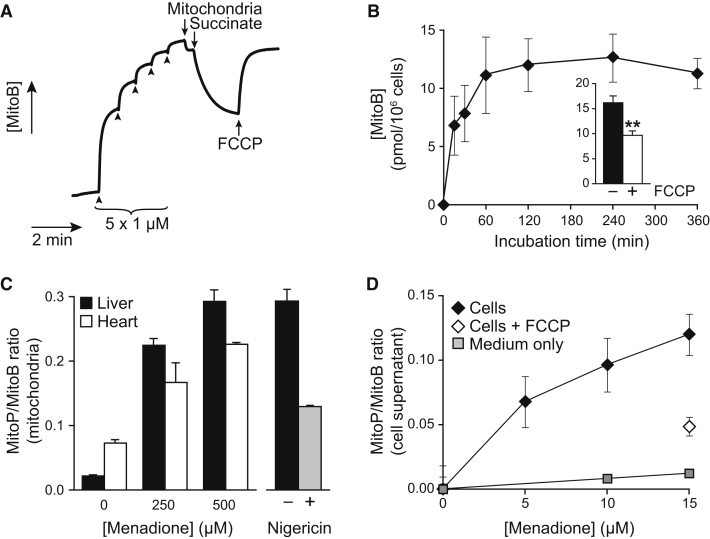
Uptake of MitoB into Energized Mitochondria and Cells, and Detection of Oxidative Stress (A) Uptake of MitoB by energized mitochondria. An electrode selective for the TPP cation was inserted in a stirred 3 ml chamber containing KCl medium (pH 7.2) at 30°C supplemented with rotenone (4 μg/ml) and nigericin (100 nM), and calibrated by five sequential additions of 1 μM MitoB (arrowheads). Mitochondria (2 mg protein/ml) were then added, followed by the respiratory substrate succinate (10 mM) and finally FCCP (500 nM). (B) Uptake of MitoB into cells. Jurkat cells (3 × 10^6^/ml) were incubated with 5 μM MitoB, and the amount of MitoB in the cell pellets was determined by LC-MS/MS. Data are means ± range of two independent experiments. (Inset) Jurkat cells (3 × 10^6^/ml) were incubated in PBS supplemented with 1 mM pyruvate and 5 μM MitoB ± 500 nM FCCP for 1 hr. Samples (950 μl) of the cell suspension were then pelleted and the MitoB content determined by LC-MS/MS. Data are means ± SD of three replicates. ^∗∗^p < 0.01 by a two-tailed Student's t test. (C) Oxidation of MitoB to MitoP within mitochondria exposed to oxidative stress. (Left) Rat liver and heart mitochondria (1 mg protein/ml) were incubated for 10 min in KCl medium (pH 7.2) at 37°C with 50 U/ml catalase and the indicated concentration of menadione. The MitoP/MitoB ratio in the mitochondrial pellet was determined by LC-MS/MS. (Right) Liver mitochondria were incubated as described above with 500 μM menadione in the presence of nigericin (100 nM). Data are means ± range of duplicate samples. (D) MitoP/MitoB ratio in the supernatant of cells exposed to oxidative stress. C2C12 cells were grown in 6-well plates seeded at 2 × 10^5^/well and incubated overnight. The cells were then supplied with fresh medium containing 5 μM MitoB, 50 U/ml catalase, and the indicated concentration of menadione and incubated for 6 hr. Controls without cells (medium only) and cells with the uncoupler FCCP (5 μM) were performed in parallel. Data are means ± SEM of three replicates (except for the medium only control, which are means ± range), and were corrected for the t = 0 value.

MitoB was rapidly taken up by mitochondria when the respiratory substrate succinate was added to generate a membrane potential (*Δψ_m_*) (Figure 4A). The accumulated MitoB was released once the *Δψ_m_* was abolished by the uncoupler carbonyl cyanide *p*-trifluoromethoxyphenylhydrazone (FCCP) (Figure 4A). MitoP was similarly accumulated and released (Figure S4). Mitochondria accumulated ∼9 nmol MitoB into a matrix volume of ∼0.5 μl/mg protein (Ross et al., 2008), giving an intramitochondrial concentration of ∼3 mM. This equates to a 3000-fold accumulation relative to the external concentration (∼1 μM), as expected for the mitochondrial uptake and binding of TPP compounds (Ross et al., 2008).

Incubation of MitoB with Jurkat cells showed rapid MitoB uptake that came to a steady state of ∼15 pmol MitoB/million cells (Figure 4B), consistent with other TPP cations (Ross et al., 2008). FCCP decreased MitoB uptake by ∼40% (Figure 4B, inset), comparable to that of [^3^H]-methylTPP (∼48%). This residual cell association of methylTPP and MitoB is caused by uptake into the cytosol due to the plasma membrane potential and to nonspecific binding (Brand, 1995, Ross et al., 2008). As intracellular methylTPP is >90% present within mitochondria, the similar effects of FCCP on MitoB uptake indicate that intracellular MitoB is predominantly mitochondrial (Ross et al., 2008).

To determine whether MitoB was converted to MitoP by H_2_O_2_ within mitochondria, we used the redox cycler menadione to induce H_2_O_2_ formation in the mitochondrial matrix (Xu and Arriaga, 2009). Control mitochondria generated negligible amounts of H_2_O_2_ but that was increased to ∼960 ± 30 nM H_2_O_2_/min/mg protein by menadione which was unaffected by 5 μM MitoB (900 ± 80 nM/min/mg protein) (means ± ranges). Incubation of mitochondria with MitoB in the presence of catalase followed by measurement of the mitochondrial MitoP/MitoB ratio gave a low value that increased in the presence of menadione (Figure 4C). The K^+^/H^+^ ionophore nigericin lowered the MitoP/MitoB ratio by ∼56% (Figure 4C) but had marginal effects on H_2_O_2_ production (-11%) and MitoB uptake (+15%). Nigericin lowers the matrix pH from ∼8.0 to 7.2 (Brand, 1995); hence the decrease in MitoP/MitoB ratio supports the enhanced reactivity of MitoB with H_2_O_2_ in the alkaline mitochondrial matrix. To assess whether MitoB could report on H_2_O_2_ production by mitochondria inside cells, we incubated C2C12 cells with MitoB for 6 hr (Figure 4D). TPP cations equilibrate rapidly with the incubation medium (Ross et al., 2008), so we measured the MitoP/MitoB ratio in the culture medium (Figure 4D). Oxidation of MitoB was negligible in cell-free medium, while cells in the presence of menadione gave a concentration-dependent increase in the MitoP/MitoB ratio (Figure 4D). FCCP decreased, but did not completely abolish MitoB oxidation, as there is still some uptake of TPP cations into the cytosol and mitochondria in its presence (Brand, 1995, Ross et al., 2008). Parallel measurements of the MitoP/MitoB ratio within the cell layer showed that it increased in response to menadione and correlated with the ratio in the medium (R^2^ > 0.95, data not shown). We conclude that the MitoP/MitoB ratio reports on mitochondrial H_2_O_2_ production within cells.

### The MitoP/MitoB Ratio Reports on Mitochondrial H_2_O_2_ Levels In Vivo

To determine if MitoB responded to mitochondrial H_2_O_2_ within living organisms, the fruit fly *Drosophila melanogaster* was used. MitoB was injected into the fly thorax (∼50 nmol MitoB/g wet weight), and no signs of toxicity were found up to 48 hr after injection. Assessment of head, thorax, and abdomen showed that MitoB distributed throughout the fly with most being in the thorax (Figure S5A). Normalizing the MitoB distribution to the wet weights of each body section showed that the MitoB concentration was 3- to 4-fold higher in the thorax compared to the head or abdomen (Figure S5B). This distribution of MitoB matched that of the mitochondrial marker enzyme citrate synthase (Magwere et al., 2006a) (Figure S5C), consistent with most mitochondria being in thoracic flight muscle, and with MitoB concentrating within mitochondria in vivo. It is difficult to confirm that a TPP compound is in mitochondria in vivo, because they rapidly redistribute on homogenization. Therefore, to demonstrate mitochondrial localization in vivo, surrogate TPP molecules are used that react with thiol proteins, decorating them with TPP moieties that can be subsequently detected on western blots (Ross et al., 2008, Porteous et al., 2010). Iodoacetamide-TPP (Porteous et al., 2010) was injected into flies, and analysis of mitochondria isolated 3 hr later showed most of the TPP bound to mitochondrial protein (Figure S5D), with negligible amounts detectable in the cytosol (data not shown).

**Figure 5:**
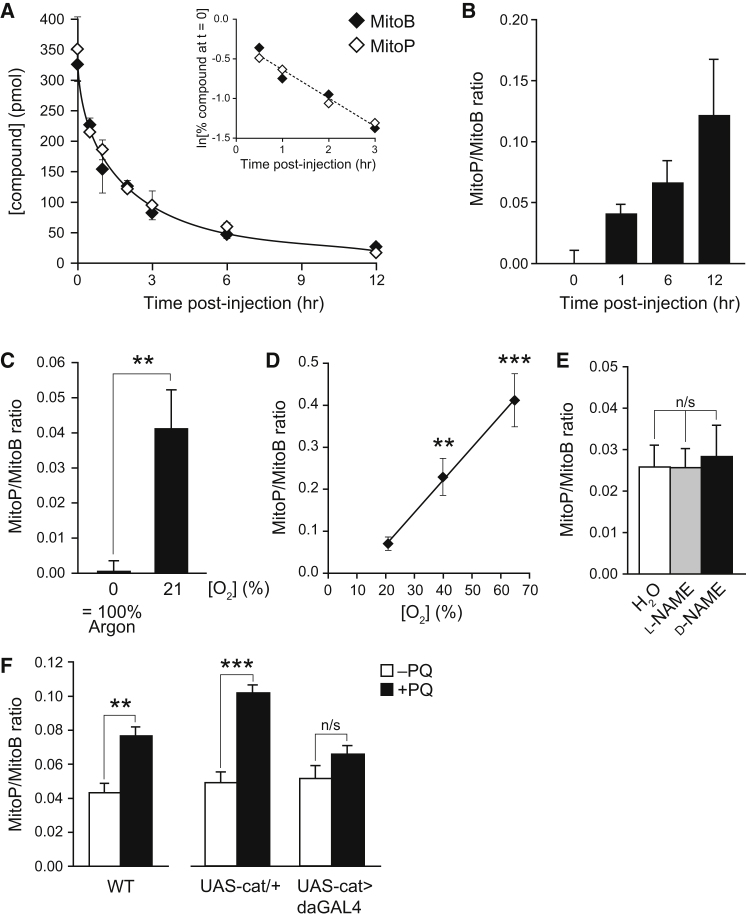
Detection of Mitochondrial H_2_O_2_ within Living Flies (A) Clearance of MitoB and MitoP from flies following injection. Groups of ten females were each injected with either MitoB or MitoP (∼75 pmol/fly). At the indicated times after injection, groups of flies were snap frozen and stored at -80°C until processing for LC-MS/MS analysis. Data are means ± SD of three replicates. The inset shows a plot of the natural logarithm of the compounds concentrations (as a percent of the amount at t = 0) over time to determine the first order rates of clearance for MitoB and MitoP. The slope of the linear trend line was -0.3556 hr^−1^ (R^2^ = 0.967). (B) Ratio of MitoP/MitoB in flies over time. Flies were injected with MitoB as in (A) and then maintained under normal aerobic conditions for the indicated time before measuring the MitoP/MitoB ratios. Data are means ± range of duplicate samples, and were corrected for the t = 0 value. (C) Effect of anoxia on the MitoP/MitoB ratio in flies. Flies that had been injected with MitoB as in (A) were incubated under anoxia (100% argon) or normoxia (21% O_2_) for 3 hr, and then the MitoP/MitoB ratio was determined. Data are means ± SEM of five replicates. (D) Effect of O_2_ concentration on the MitoP/MitoB ratio in flies. Flies that had been injected with MitoB as in (A) were incubated under normoxia (21% O_2_) or hyperoxia (40 or 65% O_2_) for 6 hr. Then the MitoP/MitoB ratio was determined. Data are means ± SEM of three to five replicates. (E) Effect of l-NAME on the MitoP/MitoB ratio within flies. Wild-type flies were pretreated with food containing water, 100 mM l-NAME, or 100 mM d-NAME for 48 hr prior to MitoB injection, and then maintained on that food for a further 3 hr. Data are means ± SD of four to five replicates. (F) Effect of paraquat on the MitoP/MitoB ratio within flies. (Left) Wild-type flies were pretreated with food ± 20 mM paraquat for 6 hr prior to MitoB injection, then maintained on that food for a further 6 hr. Data are means ± SEM of four to five replicates. (Right) Ubiquitous catalase overexpression protects against PQ toxicity. Control (UAS-cat/+) and transgenic flies overexpressing catalase (UAS-cat > daGAL4) were pretreated with food ± 20 mM paraquat for 6 hr prior to MitoB injection, then maintained on that food for a further 3 hr. Data are means ± SEM of four to five replicates. Statistical significance was determined by a two-tailed Student's t test: ^∗^p < 0.05, ^∗∗^p < 0.01, ^∗∗∗^p < 0.001.

The fates of MitoB and MitoP within flies were assessed with cohorts which were injected with ∼75 pmol compound/fly and sampled at various times. MitoB and MitoP were readily detected within flies with both compounds being eliminated over ∼12 hr with the same first-order elimination kinetics (Figure 5A). The rate constant for elimination was 0.356 hr^−1^ (Figure 5A, inset), corresponding to a half-life of ∼1.9 hr. This indicates that changes in the MitoP/MitoB ratio are due to MitoB oxidation to MitoP, not to differential excretion.

The conversion of MitoB to MitoP in flies was demonstrated by injecting MitoB and measuring the MitoP/MitoB ratio over 12 hr (Figure 5B). This ratio increased over time, demonstrating the generation of H_2_O_2_ in vivo under normal metabolic conditions. In contrast, the MitoP/MitoB ratio did not change in flies that were maintained in an anaerobic, argon atmosphere postinjection (Figure 5C), demonstrating that MitoB was only converted to MitoP in response to the H_2_O_2_ production during aerobic metabolism. To confirm that MitoB was predominantly converted to MitoP, we conducted an LC-MS/MS screen for TPP-containing compounds that accumulated in flies following MitoB injection and found that only MitoP was produced from MitoB over time (Figure S5E). Therefore we conclude that in vivo MitoB is either converted to MitoP or is excreted. Thus the increase in the MitoP/MitoB ratio within living flies is consistent with MitoB reacting with mitochondrial H_2_O_2_ during aerobic metabolism to form MitoP.

The possible application of MitoB to other animal models was established with proof-of-principle experiments in mice and with the nematode *Caenorhabditis elegans.* When worms were incubated with MitoB in the presence of paraquat, the MitoP/MitoB ratio within the worms increased (Figure S6A). When mice were infused intravenously with MitoB, both MitoB and MitoP were readily detected in the tissues and urine, and the ratio increased with time (Figures S6B-S6E). These findings are consistent with the MitoP/MitoB ratio responding to mitochondrial ROS in vivo in these important model organisms.

Confirmation that changes in the MitoP/MitoB ratio within flies was in response to alterations in mitochondrial H_2_O_2_ was secured by comparing flies maintained under normoxic conditions (21% O_2_) with those incubated under hyperoxia (40% or 65% O_2_). Hyperoxia increases mitochondrial H_2_O_2_ production because mitochondrial superoxide production is dependent on [O_2_] (Murphy, 2009). When [O_2_] was elevated, we observed a corresponding increase in the MitoP/MitoB ratio (Figure 5D). To assess whether formation of ONOO^-^ from NO and superoxide within flies may convert MitoB to MitoP, we treated flies with the NO synthase inhibitor *N*-nitro-l-arginine methyl ester (l-NAME), or with the inactive isomer d-NAME (Brown et al., 2009). Blocking NO synthesis did not significantly decrease the MitoP/MitoB ratio, either under normoxia (Figure 5E) or hyperoxia (40% O_2_, data not shown), indicating that ONOO^-^ does not contribute to MitoB oxidation in our experiments. Preadministering the redox cycler paraquat to flies before injecting MitoB significantly increased the MitoP/MitoB ratio (Figure 5F, left). Ubiquitous catalase overexpression in the cytosol using the UAS/GAL4 system enhanced catalase levels ∼6-fold at the mRNA level, as assessed by RT-qPCR (data not shown), and protected against paraquat toxicity (Figure 5F, right). Together, these data indicate that the MitoP/MitoB ratio only responds to interventions that alter mitochondrial H_2_O_2_ within the living fly.

### Mitochondrial H_2_O_2_ in *Drosophila* during Aging and Physical Activity

**Figure 6:**
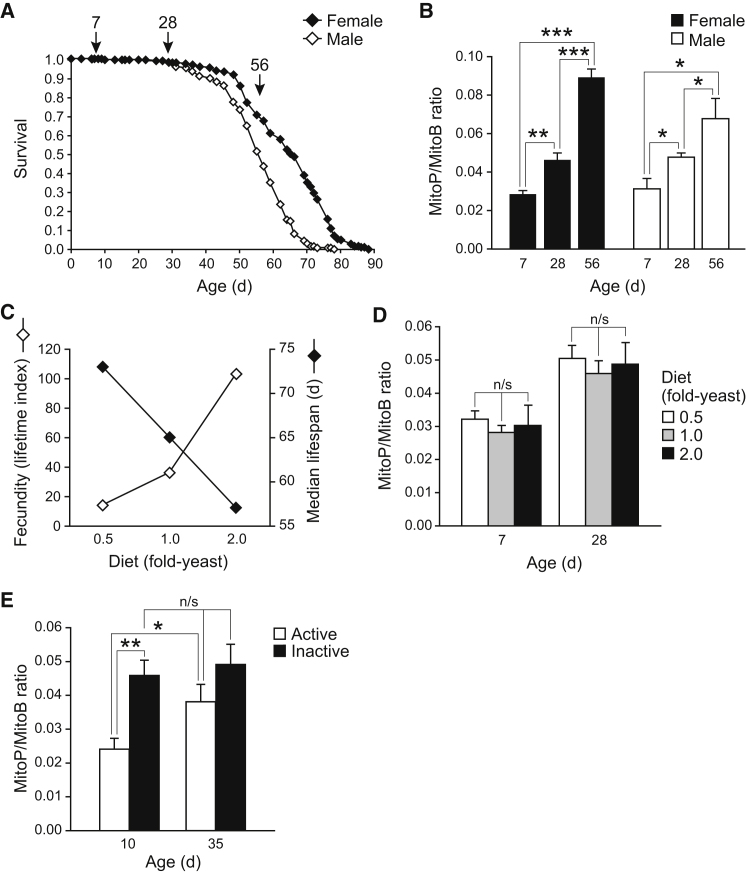
Using MitoB to Assess the Role of Mitochondrial ROS In Vivo in *Drosophila* (A) Typical life span curves for wild-type *Drosophila* under standard conditions (n = 200 per sex). (B) Effect of age on the MitoP/MitoB ratio in wild-type flies. Flies injected with MitoB were incubated for 6 hr (females) or 3 hr (males), then extracted and analyzed by LC-MS/MS. Data are means ± SEM of three to five samples. Note that due to the different incubation times in these experiments (done for technical reasons, because males are smaller and therefore injected with proportionately less MitoB), the ratio values should not be compared directly between the sexes. (C) DR affects fecundity and life span in female *Drosophila*. Data for wild-type Dahomey flies were adapted from (Grandison et al., 2009). DR was achieved by diluting the concentration of yeast provided in the food, at 0.5-, 1.0-, and 2.0-fold the standard amount. The fecundity index is a measure of cumulative egg-laying per female throughout life, which decreases under DR conditions, whereas median life span increases. (D) Effect of DR on the MitoP/MitoB ratio in wild-type female flies. Flies were maintained on the indicated diets from 2 days. Flies were injected with MitoB, incubated for 6 hr, then extracted and analyzed by LC-MS/MS. Data are means ± SEM of four to five samples. (E) Effect of activity on the MitoP/MitoB ratio in wild-type female *Drosophila*. Flies were maintained under standard “active” conditions, which allows flying and jumping, or under “inactive” conditions from day 2, where movement is spatially restricted and only walking is possible. Flies injected with MitoB were incubated for 3 hr, then extracted and analyzed by LC-MS/MS. Data are means ± SEM of four to five samples. Statistical significance was determined by a two-tailed Student's t test: n/s p > 0.05, ^∗^p < 0.05, ^∗∗^p < 0.01, ^∗∗∗^p < 0.001.

The ability to measure mitochondrial H_2_O_2_ within living flies opened the way to addressing unresolved questions about the roles of mitochondrial H_2_O_2_ in vivo. Mitochondrial oxidative damage increases with aging (Beckman and Ames, 1998), but it is unclear if this is due to an increase in ROS or to decreases in antioxidant defenses, damage repair, and turnover (Finkel, 2005, Murphy, 2009). Differences in mitochondrial H_2_O_2_ during aging were examined by comparing the MitoP/MitoB ratio in young (7-day-old), middle-aged (28-day-old) and elderly (56-day-old) flies (Figure 6A). There was an age-dependent increase in MitoP/MitoB during aging for both sexes (Figure 6B).

The increased mitochondrial H_2_O_2_ observed during aging may contribute to increased mortality, or may simply be an epiphenomenon with no functional impact (Murphy and Partridge, 2008). To address this, we used two interventions that modulate *Drosophila* life span robustly, and tested their effects on mitochondrial H_2_O_2_ levels. The first intervention was DR, which has a reproducible effect on female fecundity and life span in flies fed 0.5-, 1.0-, and 2.0-fold the standard amount of yeast in their diet (Figure 6C, data from Grandison et al., 2009). There were no alterations in the MitoP/MitoB ratio between the three diets at either 7 days or 28 days (Figure 6D), despite large alterations in median life span and fecundity. This suggests that the effects of DR in this model are not due to changes in average mitochondrial matrix H_2_O_2_ levels in vivo.

The next intervention investigated was physical activity. The median life span of flies that were free to fly and jump was decreased by 33% relative to controls maintained under spatially limited conditions that only permitted walking (Magwere et al., 2006b). We compared the MitoP/MitoB ratio of active and inactive flies, and found that young (10-day-old) inactive flies had a significantly higher MitoP/MitoB ratio than did active controls (Figure 6E). The difference in the MitoP/MitoB ratio between active and inactive older (35-day-old) flies was not statistically significantly (Figure 6E), consistent with the decline in physical activity with age (Magwere et al., 2006b).

Together these data indicate that average mitochondrial H_2_O_2_ levels increase with aging. However, DR, an intervention that robustly alters life span, has no impact on overall mitochondrial H_2_O_2_ levels. Conversely, increasing fly physical activity decreases life span (Magwere et al., 2006b), despite decreasing average mitochondrial H_2_O_2_ levels. Therefore, the changes in life span in flies due to these interventions are not predominantly due to alteration in the average levels of mitochondrial H_2_O_2_.

## Discussion

Changes in the concentration of mitochondrial matrix H_2_O_2_ have been assumed to play a central role in many pathologies and pathways in vivo. However, it has not been possible to test these hypotheses directly until now, due to the difficulty in measuring mitochondrial ROS levels in vivo. Inferences based on ROS fluxes from isolated mitochondria in vitro cannot be extrapolated to the in vivo situation (Murphy, 2009). This was confirmed, as the significant differences in mitochondrial [H_2_O_2_] in vivo due to physical activity that we found here were not seen in vitro for mitochondria isolated from active and inactive flies (Magwere et al., 2006b). Likewise, measurements of oxidative damage are also difficult to relate to ROS flux without detailed knowledge of the changes in defense, repair, and damage turnover pathways. Furthermore, H_2_O_2_ acts as a direct redox signal independent of the accumulation of oxidative damage (Janssen-Heininger et al., 2008). To address this unmet need, we developed a mitochondria-targeted mass spectrometry H_2_O_2_ probe, MitoB. This approach represents a significant development that will complement current methods to measure mitochondrial ROS using fluorescent probes (Belousov et al., 2006, Dickinson and Chang, 2008), but which are only applicable to cultured cells or to optically amenable organisms such as zebrafish and *C. elegans.* Previous approaches to assess free radical production in vivo have used electron paramagnetic resonance (EPR) spin traps in conjunction with LC-MS/MS (e.g., Reis et al., 2009, Yue Qian et al., 2005). However, the methodology described here is a significant improvement, due to the *Δψ_m_*-dependent uptake of the TPP compounds into mitochondria in vivo, the selectivity of the chemical reaction of MitoB with H_2_O_2_, the further selectivity for mitochondrial H_2_O_2_ due to the increased pH of the matrix, the chemical and biological stability of MitoP, the increased mass spectrometric sensitivity due to the inherent positive charge of the TPP moiety, the ratiometric analysis, and the ability to compare and quantify changes in MitoP and MitoB relative to their deuterated ISs. The methodology outlined here measures the average mitochondrial matrix H_2_O_2_ concentration throughout the whole organism, and complements optical measurements of the site and kinetics of ROS production.

In the present study, we have focused on assessing mitochondrial H_2_O_2_ in whole flies following injection of MitoB. However, we also provide proof of principle that this approach can be extended to other experimental models and modes of administration. Although mitochondrial ROS in cells can be assessed using fluorescent probes, its quantification, repeated assessment, and comparison of multiple samples can be challenging. The equilibration of the MitoP/MitoB with the extracellular medium facilitates measurement of mitochondrial H_2_O_2_ within cells over time in multiple incubations. MitoB was taken up into the nematode *C. elegans*, and the MitoP/MitoB ratio was responsive to increased oxidative stress. In mice, intravenous infusion led to the rapid uptake of MitoB from the circulation into the tissues, as expected for TPP compounds (Porteous et al., 2010). The MitoP/MitoB ratio within mouse tissues was easily measured, and its increase over time was consistent with it responding to mitochondrial H_2_O_2_. The MitoP/MitoB ratio in mouse urine was also readily quantified, raising the prospect of noninvasive assessments of whole-body mitochondrial ROS.

The conversion of MitoB to MitoP within *Drosophila* is most likely due to mitochondrial H_2_O_2_. As MitoB is also converted to MitoP by reaction with ONOO^-^, conditions that lead to ONOO^-^ formation within mitochondria might also form MitoP. A single NO synthase is expressed in *Drosophila* that produces NO during development (Regulski and Tully, 1995). However, as there is no indication of ONOO^-^ formation in *Drosophila*, and inhibiting NO synthase with l-NAME did not affect MitoB oxidation, it is reasonable to assume that the changes to MitoB are due to H_2_O_2_.

Following its injection into the fly thorax, MitoB distributes rapidly throughout the fly with the extent of distribution between the head, thorax, and abdomen consistent with its uptake into mitochondria. As most of a fly's mitochondria are in the thoracic flight muscle, this approach will predominantly report on these. Use of a visualizable TPP compound as a MitoB surrogate indicated that most injected TPP compound inside flies was present within mitochondria. Together these data suggest that most MitoB and MitoP within the fly is present in mitochondria. This is supported by calculations in the Supplemental Information indicating that most MitoB within flies (∼90%) is present within cells and that nearly all of this (∼98%) is present within mitochondria. As the reaction of MitoB with H_2_O_2_ is second order, the several-hundred-fold concentration of MitoB within mitochondria will lead to a similar fold enhancement of its reaction rate with mitochondrial versus cytoplasmic H_2_O_2_. Furthermore, MitoB is ∼4-fold more reactive with H_2_O_2_ at the mitochondrial matrix pH relative to that in the cytosol. The only product of MitoB detected was MitoP, and the clearance rates of MitoB and MitoP from flies were indistinguishable. The MitoP/MitoB ratio increased over time and with interventions known to elevate mitochondrial H_2_O_2_, whereas overexpression of catalase decreased the effect of paraquat on the MitoP/MitoB ratio. While the localization and reactivity of MitoB mean that it responds to mitochondrial H_2_O_2_, mitochondria are not necessarily the source, as H_2_O_2_ can readily diffuse into mitochondria from elsewhere. This approach reports on the whole organism over time, and thus gives an average mitochondrial H_2_O_2_ concentration for the whole organism: changes in mitochondrial H_2_O_2_ that are localized or transient may be difficult to detect. We conclude that alterations in the MitoP/MitoB ratio within the fly reflect the average mitochondrial H_2_O_2_ concentration inside living *Drosophila*.

As the uptake of MitoB into mitochondria is dependent on *Δψ_m_*, changes in this potential will influence the mitochondrial concentration of MitoB. However, alterations to *Δψ_m_* will not distort the assessment of mitochondrial H_2_O_2_. This is because the MitoP/MitoB ratio is measured, so any differences in MitoP formation due to alterations in the uptake of MitoB caused by changes in *Δψ_m_* are normalized to the parallel changes in MitoB, which is accumulated by mitochondria to the same extent as MitoP and also eliminated from the body with identical kinetics. Furthermore, once *Δψ_m_* decreases below about ∼120 mV, ATP synthesis is no longer possible, and reversal of the ATP synthase will attenuate further decreases. Consequently *Δψ_m_* is unlikely to deviate from the range 120-160 mV in vivo. The effect of such a change in *Δψ_m_* on the proportion of intracellular MitoB within mitochondria is very small and can be determined from Equation S5: changing *Δψ_m_* from 120 mV to 160 mV alters the percent of intracellular MitoB that is mitochondrial from 97% to 99%. Thus any change in MitoB uptake within flies in vivo does not impact on the MitoP/MitoB ratio. This can be confirmed directly by comparing MitoB content with the wide range of MitoP/MitoB ratios that occur on maintenance of flies at different percent O_2_ where no correlation (R^2^ ∼0.2) was found between the MitoP/MitoB ratio and the amount of MitoB in the fly (Figure S7A). Similarly, a plot of MitoP/MitoB against the amount of MitoB in the fly under less extreme conditions (Figure S7B) also showed no correlation (R^2^ ∼0.1). Therefore alterations in *Δψ_m_* or MitoB uptake do not distort the ability of the MitoP/MitoB ratio to report on changes in mitochondrial H_2_O_2_ in vivo.

Prior to this work, calculation of the absolute concentration of mitochondrial H_2_O_2_ in vivo has proven impossible. Therefore it is significant that the MitoP/MitoB ratio can be used to calculate the average concentration of H_2_O_2_ inside mitochondria within living flies. The rationale and equations for deriving mitochondrial [H_2_O_2_] are developed in the Supplemental Information. To summarize, the MitoP/MitoB ratio can give the mitochondrial [H_2_O_2_] within the fly provided two assumptions are made: that conversion of MitoB to MitoP only occurs to any significant extent due to direct reaction with H_2_O_2_ inside mitochondria. This assumption is justified for three reasons: (1) most (∼88%) of the MitoB within the fly is mitochondrial, (2) the several-hundred-fold concentration of MitoB within mitochondria will lead to a similar fold enhancement of its second-order reaction with H_2_O_2_, (3) and within the mitochondrial matrix MitoB has an ∼4-fold greater rate of reaction with H_2_O_2_ than elsewhere. The second assumption is that MitoP/MitoB equilibrates throughout the fly rapidly compared to the 3-6 hr duration of the experiments. This is reasonable, as conversion of MitoB to MitoP by H_2_O_2_ is slow (3.8 M^−1^s^−1^ at 25°C, pH 8.0), while the distribution of TPP cations equilibrates over minutes in cells (Ross et al., 2008) and in mice (Porteous et al., 2010). This is supported by the rapid uptake and release of MitoB by mitochondria and cells, and by its distribution throughout flies. As described in the Supplemental Information, these assumptions lead to Equation 1, where the mitochondrial H_2_O_2_ concentration, [*H_2_O_2_*]*_mito_*, is given in terms of the whole-fly MitoP/MitoB ratio, the duration of the MitoB incubation (*t*) in hours, the second-order rate constant for the reaction of MitoB with H_2_O_2_ within mitochondria (*k_2_* = 13.68 × 10^3^ M^−1^hr^−1^ at pH 8.0, 25°C), and the ratio of the MitoB concentration within mitochondria to that in the whole fly (γ). The value for γ derived in the Supplemental Information for our experiments is 3.26.

(1) $${\left[H_{2}O_{2}\right]}_{mito}=\frac{1}{\gamma k_{2}t}\log_{e}\left(\frac{{\left[MitoP\right]}_{total}}{{\left[MitoB\right]}_{total}}+1\right)$$

Equation 1 enables us to go from the measured MitoP/MitoB ratio to the [*H_2_O_2_*]*_mito_*, with parameters derived from experimentally accessible values. Applying Equation 1 to the data from Figure 5, Figure 6 gives values for [*H_2_O_2_*]*_mito_* that are shown in Table 1. The values for young (7-day-old) female flies maintained under standard conditions measured in a number of separate experiments gave [*H_2_O_2_*]*_mito_* in the range 104-250 nM with an average of 177 ± 27 nM (Table 1). Hyperoxia at 40% O_2_ and 65% O_2_ increases [*H_2_O_2_*]*_mito_* to ∼764 nM and ∼1271 nM, respectively. Treatment with paraquat increases [*H_2_O_2_*]*_mito_* to ∼276 nM. There are no direct in vivo measurements of [*H_2_O_2_*]*_mito_* in the literature. The [H_2_O_2_] within aerobically grown *Escherichia coli* is 100-200 nM, based on the equilibration between intracellular and extracellular H_2_O_2_ (Gonzalez-Flecha and Demple, 1995). This suggests that an average [*H_2_O_2_*]*_mito_* in vivo of ∼177 nM is biologically plausible. However, it is vital that our estimates are tested using orthogonal techniques, and that the robustness of the assumptions used in deriving Equation 1 is continually reassessed and the predicted values altered accordingly.

**Table 1:** Concentration of H_2_O_2_ within Mitochondria in Living Flies

Condition		Age (Days)	MitoP/MitoB Ratio	Mitochondrial [H_2_O_2_] (nM)
Normal	Females	7	0.048 ± 0.008 (13)	177 ± 27 (13)
Hyperoxia	21% O_2_	7	0.069 ± 0.016 (5)	250 ± 56 (5)
	40% O_2_	7	0.229 ± 0.044 (3)	764 ± 134 (3)^∗∗^
	65% O_2_	7	0.411 ± 0.063 (5)	1271 ± 172 (5)^∗∗∗^
Paraquat	-	7	0.043 ± 0.005 (4)	158 ± 19 (4)
	+	7	0.077 ± 0.005 (5)	276 ± 16 (5)^∗∗^
Aging	Young	7	0.028 ± 0.002 (4)	104 ± 8 (4)
	Middle-aged	28	0.046 ± 0.004 (4)	168 ± 13 (4)^∗∗^
	Old	56	0.089 ± 0.004 (3)	318 ± 15 (3)^∗∗∗^
Activity	Active	10	0.024 ± 0.003 (5)	178 ± 24 (5)
	Inactive	10	0.046 ± 0.005 (5)	335 ± 37 (5)^∗∗^

The MitoP/MitoB ratios shown are from the experiments described in Figure 5, Figure 6. The mitochondrial [H_2_O_2_] values were calculated from the MitoP/MitoB ratios using Equation 1. Data are means ± SEM for n independent experiments. Statistical significance was determined relative to the appropriate control cohort of flies for each intervention by a two-tailed Student's t test: ^∗^p < 0.05, ^∗∗^p < 0.01, ^∗∗∗^p < 0.001.

The development of this ratiometric mass spectrometric technique to assess mitochondrial H_2_O_2_ in vivo enabled us to investigate the role of mitochondrial H_2_O_2_ during aging in *Drosophila*. Oxidative damage is known to increase with aging in *Drosophila* and has been widely speculated to be a causal factor in normal aging. However, the link between the concentration of ROS such as H_2_O_2_ and the accumulation of damage in aging is not direct, because oxidative damage to biomolecules is a combination of the degrees of damage, protection, repair, and clearance. In addition, H_2_O_2_ can act as a redox signal independently of oxidative damage (Balaban et al., 2005, Murphy, 2009). To clarify these matters, we looked at [*H_2_O_2_*]*_mito_* during normal aging and found a significant increase in [*H_2_O_2_*]*_mito_* in both male and female flies corresponding to an ∼2- to 3-fold increase by 56 days of age (Table 1). This is the first direct demonstration that [*H_2_O_2_*]*_mito_* increases with aging. However, DR did not affect [*H_2_O_2_*]*_mito_*, despite increasing life span and decreasing oxidative damage (Jacobson et al., 2010, Zheng et al., 2005). Furthermore, when physical activity was minimized in young 10-day-old flies, [*H_2_O_2_*]*_mito_* nearly doubled (Table 1), even though the decreased physical activity led to an increase in life span and a decrease in oxidative damage (Magwere et al., 2006b). These findings do not mean that changes in [*H_2_O_2_*]*_mito_* have no influence on aging, as changes in [*H_2_O_2_*]*_mito_* independent of other factors may well affect life span (e.g., Schriner et al., 2005). Furthermore, it is important to point out that our approach measures a weighted average of mitochondrial [H_2_O_2_] throughout the whole organism, and due to the greater uptake of MitoB into thoracic muscle mitochondria, our measurements are dominated by changes in this subset of mitochondria. It may be that localized changes in mitochondrial [H_2_O_2_] in particular organs or in small populations of cells, for example in particular neurons, may be critical for the aging process but will not be detected by our approach. Even so, we can conclude that any causal link between the weighted average of mitochondrial [H_2_O_2_] (predominantly thoracic muscle mitochondria) and aging in the two models investigated here is either absent or masked by changes in other processes that also determine aging.

Both DR and preventing physical activity led to a decrease in the accumulation of oxidative damage (Jacobson et al., 2010, Magwere et al., 2006b, Zheng et al., 2005), despite their very different effects on [*H_2_O_2_*]*_mito_*. Thus the decreased accumulation of oxidative damage with aging caused by these two interventions is not caused by changing [*H_2_O_2_*]*_mito_*. Instead, the changes in accumulation of oxidative damage are likely to be due to changes in protection, damage repair, and turnover, rather than to increases in ROS levels. Thus DR may act by upregulating damage prevention, repair, and turnover processes, while the elevation to damage that occurs during increased physical activity may be a consequence of diverting resources, such as the ATP/ADP ratio, away from these processes. Together these findings point toward a greater role for damage prevention, repair, and turnover compared to ROS levels in the accumulation of oxidative damage during aging.

The increase in [*H_2_O_2_*]*_mito_* caused by physical inactivity is particularly informative, as it is often assumed that mitochondrial ROS production should correlate with oxygen consumption. In fact, studies with isolated mitochondria clearly predict the opposite (Murphy, 2009). As mitochondria make less ATP and consequently consume less oxygen, mitochondrial H_2_O_2_ production should increase due to the elevated *Δψ_m_*, the more reduced electron transport chain, and the higher O_2_ concentration within mitochondria (Murphy, 2009). Therefore this first demonstration that mitochondrial H_2_O_2_ in vivo is increased by decreasing physical activity is in accordance with our understanding of how mitochondria produce ROS. We acknowledge that there may still be an overall increase in ROS production during intensive exercise due to extracellular and inflammatory processes. A corollary of this finding is that the increased [*H_2_O_2_*]*_mito_* seen on aging may simply be a consequence of decreased physical activity.

To summarize, this work demonstrates that it is possible to measure the levels of H_2_O_2_ within mitochondria in living organisms and thereby to test directly hypotheses about the roles of mitochondrial H_2_O_2_ in biological processes. This approach is not limited to H_2_O_2_: many other ROS can be measured by adjusting the chemistry of the reporting group attached to the TPP moiety. Currently we are developing probes to assess other mitochondrial ROS, and it is also possible to design similar probes targeted to other cellular compartments. MitoB may be the first in a family of mass spectrometry ROS probes that combine the specificity of organelle targeting in vivo and the chemical selectivity of reactive moieties with the sensitivity of analysis by tandem mass spectrometry, and thereby act as exogenous “biomarkers” that allow reactive biological molecules to leave a recognizable footprint on the probe molecule. Here we used this approach to show that during increased physical activity the mitochondrial H_2_O_2_ concentration decreases, while during aging the average mitochondrial H_2_O_2_ concentration increases, perhaps as a consequence of decreased physical activity. However, two interventions that altered life span in flies, DR and decreased physical activity, did not do so by decreasing overall mitochondrial matrix H_2_O_2_. Together these findings point toward a greater role for alterations to damage prevention, repair, and turnover compared to ROS levels as the mechanisms by which DR and exercise affect life span in *Drosophila*.

## Experimental Procedures

### Synthesis and In Vitro Analysis of MitoB and MitoP

The syntheses of MitoB, MitoP, the ISs *d_15_*-MitoB and *d_15_*-MitoP, and the methods used to characterize their reaction in vitro are described in the Supplemental Information.

### Cell Culture and Experiments

Cells were cultured at 37°C under humidified 95% air/5% CO_2_ in medium supplemented with penicillin (100 U/ml) and streptomycin (100 μg/ml). Jurkat cells were grown in RPMI medium (Invitrogen) supplemented with 10% fetal calf serum (FCS), and cells were maintained at 0.2-2 × 10^6^ cells/ml. C2C12 cells were maintained at subconfluence in Dulbecco's modified Eagle's medium (DMEM; Invitrogen) supplemented with 10% FCS.

### Fly Culture and Experiments

The *white* Dahomey strain of *D. melanogaster* (negative for *Wolbachia*) was used as the wild-type background and maintained as described in the Supplemental Information. MitoB was injected into flies using a custom-built microinjector, consisting of a pedal-operated motor and Hamilton syringe connected to a glass microinjection needle via mineral oil-filled tubing. The needles were prepared from glass capillaries (1.0 mm outer diameter; Harvard Apparatus) using a Flaming-Brown micropipette puller. A 1 mM MitoB solution was prepared in fly Ringer's solution (3 mM CaCl_2_, 182 mM KCl, 46 mM NaCl, 10 mM Tris base [pH 7.2 HCl]) supplemented with 1% (v/v) blue food dye (FD&C Blue No.1) to visualize successful MitoB delivery. The injection volume was ∼75 nl per female fly (∼1.5 mg wet weight) and ∼45 nl per male fly (∼0.85 mg wet weight) to achieve a final MitoB dose of 50 pmol/mg. Flies (in batches of ten) were rendered immobile with CO_2_ and were injected into the thorax (left pteropleurite). This site was selected because the target consists principally of fly muscle, with minimal risk of damaging any internal structures/organs. The injection process was not harmful, as 7-day-old flies mock injected with Ringer's alone showed >99% survival after 10 days (data not shown, n > 400). The amount of MitoB injected was not toxic to the flies, with 100% survival after 48 hr (data not shown, n = 60).

### Extraction of MitoB and MitoP from Flies for LC-MS/MS Analysis

To extract MitoB and MitoP from flies, groups of ten were homogenized in a 1.5 ml eppendorf tube using a fitted pestle plunger in 200 μl 95% ACN/0.1% FA. The homogenate was spiked with IS (50 pmol each of *d_15_*-MitoB and *d_15_*-MitoP), vortexed (5 s), and pulse centrifuged to collect debris. The supernatant was transferred to a fresh tube and the fly pellet extracted with a further 200 μl 95% ACN/0.1% FA, which was pooled with the first extraction. The supernatants were then centrifuged (10 min at 16,000 × *g*), filtered through a 0.22 μm PVDF filter (Millex, from Millipore) into a fresh eppendorf tube, and dried under vacuum using a Savant SpeedVac (∼1-2 hr). The dried samples were resuspended in 100 μl 20% ACN/0.1% FA by vortexing for 5 min. Finally, the samples were centrifuged (10 min at 16,000 × *g*), and 80 μl was transferred to an autosampler vial (1.5 ml silanized, from Chromacol).

Samples were then analyzed by LC-MS/MS with multiple reaction monitoring (MRM) in positive ion mode using a Waters Quattro Ultima triple quadrupole mass spectrometer attached to a binary pump (model 1585; Jasco) and an HTC-PAL autosampler (CTC-Analytics) as described in the Supplemental Information. For all experiments, a standard curve was prepared and processed in parallel using the appropriate biological material or buffer spiked with both *d_15_*-MitoB and *d_15_*-MitoP ISs and various amounts of MitoB or MitoP. Standard curves for the response of MitoB or MitoP relative to its deuterated IS against concentration were linear over the range 1-1000 pmol with R^2^ routinely >0.99 (see Figures 3B and 3C).

## Supplemental Information

Document S1. Seven Figures, Supplemental Calculations, Supplemental Experimental Procedures, and Supplemental References
