# Supplementary material for: Measurement of H2O2 within Living Drosophila during Aging Using a Ratiometric Mass Spectrometry Probe Targeted to the Mitochondrial Matrix
Source: Cell Metab. 2011 Mar 2;13(3):340–50. doi: 10.1016/j.cmet.2011.02.003 (PMC4413513; doi:10.1016/j.cmet.2011.02.003)
Supplement: Document S1. Seven Figures, Supplemental Calculations, Supplemental Experimental Procedures, and Supplemental References [file mmc1.pdf]

Cell Metabolism, Volume *13*

Supplemental Information

Measurement of H_2O_2 within Living *Drosophila* during Aging Using a Ratiometric Mass Spectrometry Probe Targeted to the Mitochondrial Matrix

Helena M. Cochemé, Caroline Quin, Stephen J. McQuaker, Filipe Cabreiro, Angela Logan, Tracy A. Prime, Irina Abakumova, Jigna V. Patel, Ian M. Fearnley, Andrew M. James, Carolyn M. Porteous, Robin A.J. Smith, Saima Saeed, Jane E. Carré, Mervyn Singer, David Gems, Richard C. Hartley, Linda Partridge, and Michael P. Murphy

Inventory of Supplemental Information

Figure S1 relates to Figure 1

Figure S2 relates to Figure 2

Figure S3 relates to Figure 3

Figure S4 relates to Figure 4

Figure S5 relates to Figure 5

Figure S6 relates to Figure 6

Figure S7 relates to Table 1

Figure S1

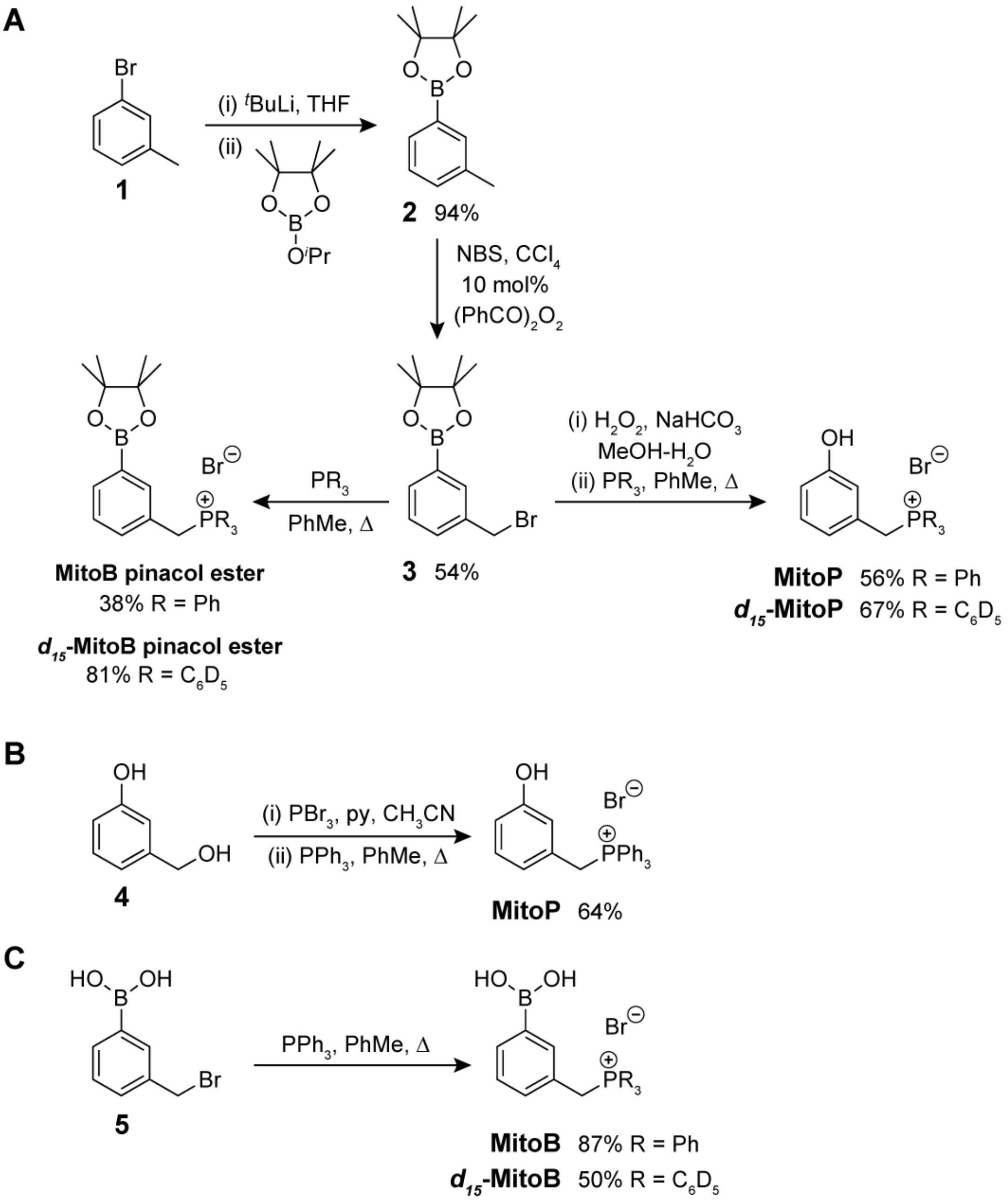

Figure S1. Synthesis of MitoB Pinacol Ester, MitoP and MitoB

(A) Preparation of MitoP and MitoB pinacol ester and their deuterated analogues.
(B) More efficient route to MitoP for future work.
(C) Preparation of MitoB and its deuterated analogue.

Figure S2

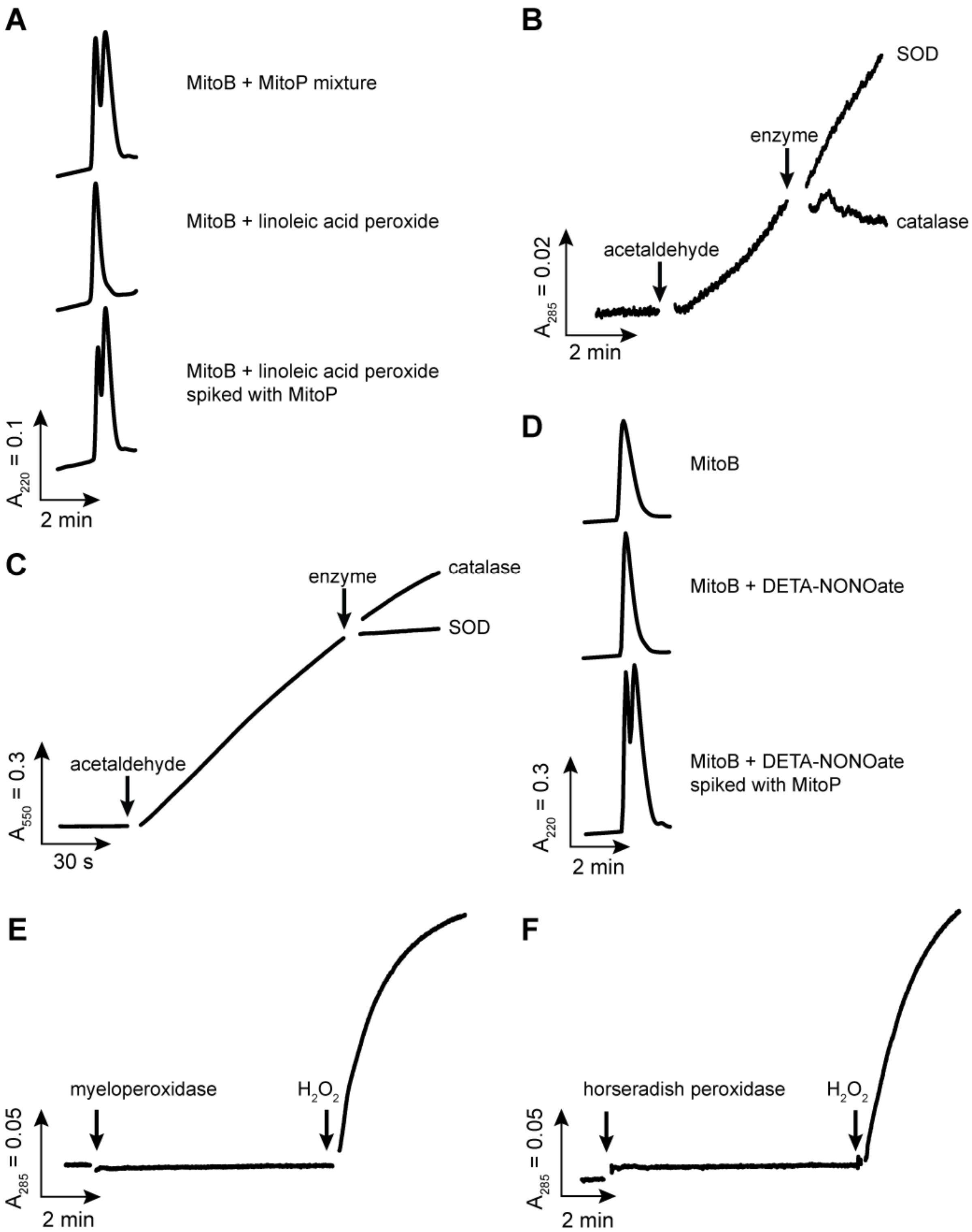
A
MitoB + MitoP mixture
MitoB + linoleic acid peroxide
MitoB + linoleic acid peroxide spiked with MitoP
A_{220} = 0.1
2 min
B
SOD
enzyme
catalase
acetaldehyde
A_{285} = 0.02
2 min
C
catalase
enzyme
SOD
acetaldehyde
A_{550} = 0.3
30 s
D
MitoB
MitoB + DETA-NONOate
MitoB + DETA-NONOate spiked with MitoP
A_{220} = 0.3
2 min
E
myeloperoxidase
H_2O_2
A_{285} = 0.05
2 min
F
horseradish peroxidase
H_2O_2
A_{285} = 0.05
2 min

Figure S2. MitoB Does Not React with Linoleic Acid Peroxide, Superoxide, Nitric Oxide or Peroxidases

(A) Lack of reaction of MitoB with linoleic acid peroxide. Linoleic acid peroxide (500 μM) was incubated with 100 μM MitoB in a 1:1 mixture of KCl medium (pH 8) and ethanol at 37ºC for 1 h under an argon atmosphere. Samples were filtered (0.22 μm), diluted 1:4 in water, and 50 μl of the mixture was separated by RP-HPLC. This was compared with a mixture of 100 μM MitoB + 100 μM MitoP (top), and with a mixture of linoleic acid peroxide (500 μM) that had been incubated with 100 μM MitoB for 1 h and then spiked with 100 μM MitoP (bottom).
(B) Oxidation of MitoB to MitoP during superoxide generation by xanthine oxidase (XO). Matched cuvettes containing KCl medium (pH 8.0) and 32 mU/ml XO were set up. MitoB (100 μM) was added to the sample cuvette and its oxidation measured at 285 nm. The XO substrate acetaldehyde (5 mM), SOD (100 U/ml) and catalase (400 U/ml) were added to both cuvettes where indicated.
(C) Assessment of superoxide production by the XO system during MitoB oxidation. Matched cuvettes containing KCl medium (pH 8.0) and 32 mU/ml XO were set up. MitoB (100 μM) and ferricytochrome *c* (50 μM) were added to the sample cuvette, and the reduction of ferricytochrome *c* was followed at 550 nm. Where indicated acetaldehyde (5 mM), SOD (100 U/ml) or catalase (400 U/ml) were added to both cuvettes. Addition of superoxide dismutase (SOD) did not markedly affect MitoB oxidation, despite degrading superoxide, while the H_2O_2-degrading enzyme catalase did prevent MitoB oxidation. Therefore, MitoB does not react with superoxide, but with the H_2O_2 produced from its dismutation. Note that the addition of SOD is expected to have little effect on the H_2O_2 concentration because of the high spontaneous rate of superoxide dismutation under these conditions.
(D) Lack of reaction of MitoB with nitric oxide (NO). MitoB (100 μM) was incubated ± the NO donor 3,3-bis(aminoethyl)-1-hydroxy-2-oxo-1-triazene (DETA-NONOate) (500 μM) in KCl medium (pH 7.2) at 37ºC for 1 h under an argon atmosphere, which leads to persistent free NO concentrations of > 2 μM (Dahm et al., 2006). A sample (50 μl) of the mixture was then separated by RP-HPLC. These were compared with a mixture of DETA-NONOate (500 μM) that had been incubated with 100 μM MitoB for 1 h and then spiked with 100 μM MitoP (bottom).
(E) Lack of oxidation of MitoB to MitoP by myeloperoxidase. Matched 1 ml cuvettes containing KCl medium (pH 7.2) and either MitoB (100 μM) or carrier were set up and the oxidation of MitoB to MitoP was measured at 285 nm. Where indicated myeloperoxidase (1 U; Sigma) was added followed by 5 mM H_2O_2 to confirm that MitoB was oxidizable under these conditions.
(F) Lack of oxidation of MitoB to MitoP by horseradish peroxidase. Matched 1 ml cuvettes containing KCl medium (pH 7.2) and either MitoB (100 μM) or carrier were set up and the oxidation of MitoB to MitoP was measured at 285 nm. Where indicated horseradish peroxidase (5 U; Sigma) was added to both cuvettes followed by 5 mM H_2O_2 to confirm that MitoB was oxidizable under these conditions.

Figure S3

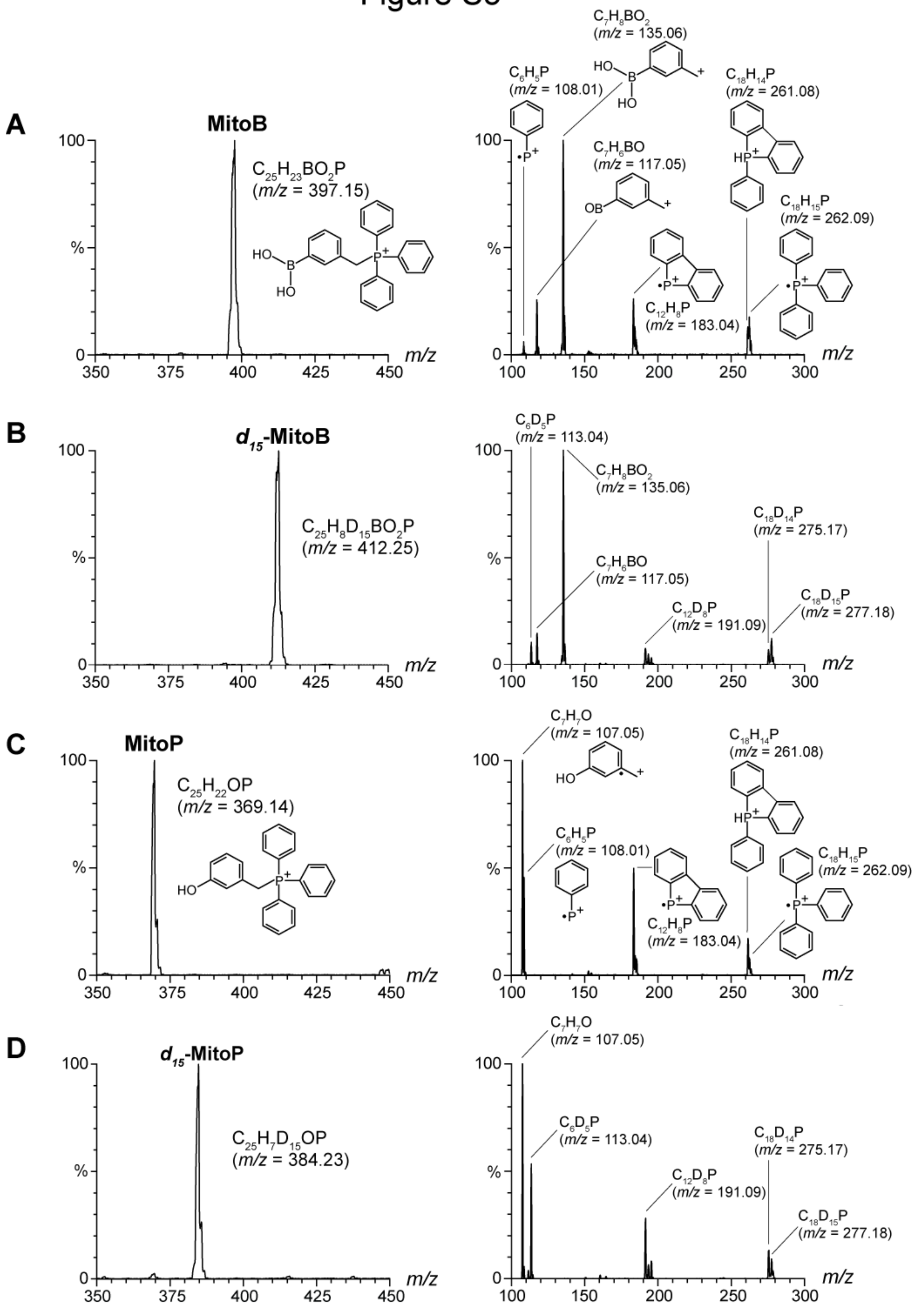

PARENT COMPOUND → DAUGHTER IONS

Figure S3. Fragmentation of MitoB and MitoP by Tandem Mass Spectrometry

Solutions of 1 µM MitoP in 50% ACN at 5 µl/min or 1 µM MitoB boronic pinacol ester in 20% ACN/0.1% FA at 2 µl/min were directly infused into the mass spectrometer. The pinacol esters rapidly and completely hydrolyzed to MitoB under these conditions. The fragmentation of MitoB (*m/z* = 397.15) is shown in (A) and gave prominent daughter ions at *m/z*: 108, 117, 135, 183, 261 and 262. Fragmentation of d_{15}-MitoB (*m/z* = 412.25), shown in (B), gave prominent daughter ions of 113, 117, 135, 191, 275 and 277. Fragmentation of MitoP (*m/z* = 369.14) is shown in (C) and gave prominent daughter ions at *m/z*: 107, 108, 183, 261 and 262. Fragmentation of d_{15}-MitoP (*m/z* = 384.23), shown in (D), gave prominent daughter ions at *m/z*: 107, 113, 191, 275 and 277. Published fragmentation patterns for alkylTPP ions (Claereboudt et al., 1993; Denekamp et al., 1999; Denekamp et al., 2003) and the presence of the 15 deuterons solely on the TPP indicated that the fragments found in both MitoB and MitoP were: $C_6H_5(D_5)P$ = 108.01 (113.04); $C_{12}H_8(D_8)P$ = 183.04 (191.09); $C_{18}H_{14}(D_{14})P$ = 261.08 (275.17); and $C_{18}H_{15}(D_{15})P$ = 262.09 (277.18). The *m/z* 117 and 135 fragments, which only arose from MitoB and were not altered by deuteration, are tentatively assigned to C_7H_6BO and $C_7H_8O_2B$ respectively. The *m/z* 107 fragment, which only arose from MitoP and was not altered by deuteration, is tentatively assigned to the C_7H_8O.

Figure S4

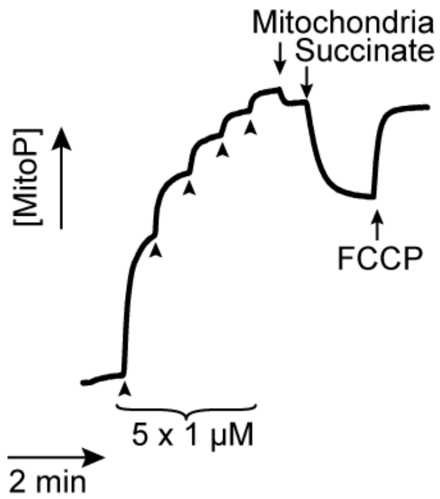

Figure S4. Uptake of MitoP by Energized Mitochondria
An electrode selective for the TPP cation was inserted in a stirred 3 ml chamber containing KCl medium (pH 7.2) supplemented with rotenone (4 µg/ml) and nigericin (100 nM) at 30ºC and calibrated by five sequential additions of 1 µM MitoP (arrowheads). Rat liver mitochondria (2 mg protein/ml) were then added, followed by succinate (10 mM) and the uncoupler FCCP (500 nM).

Figure S5

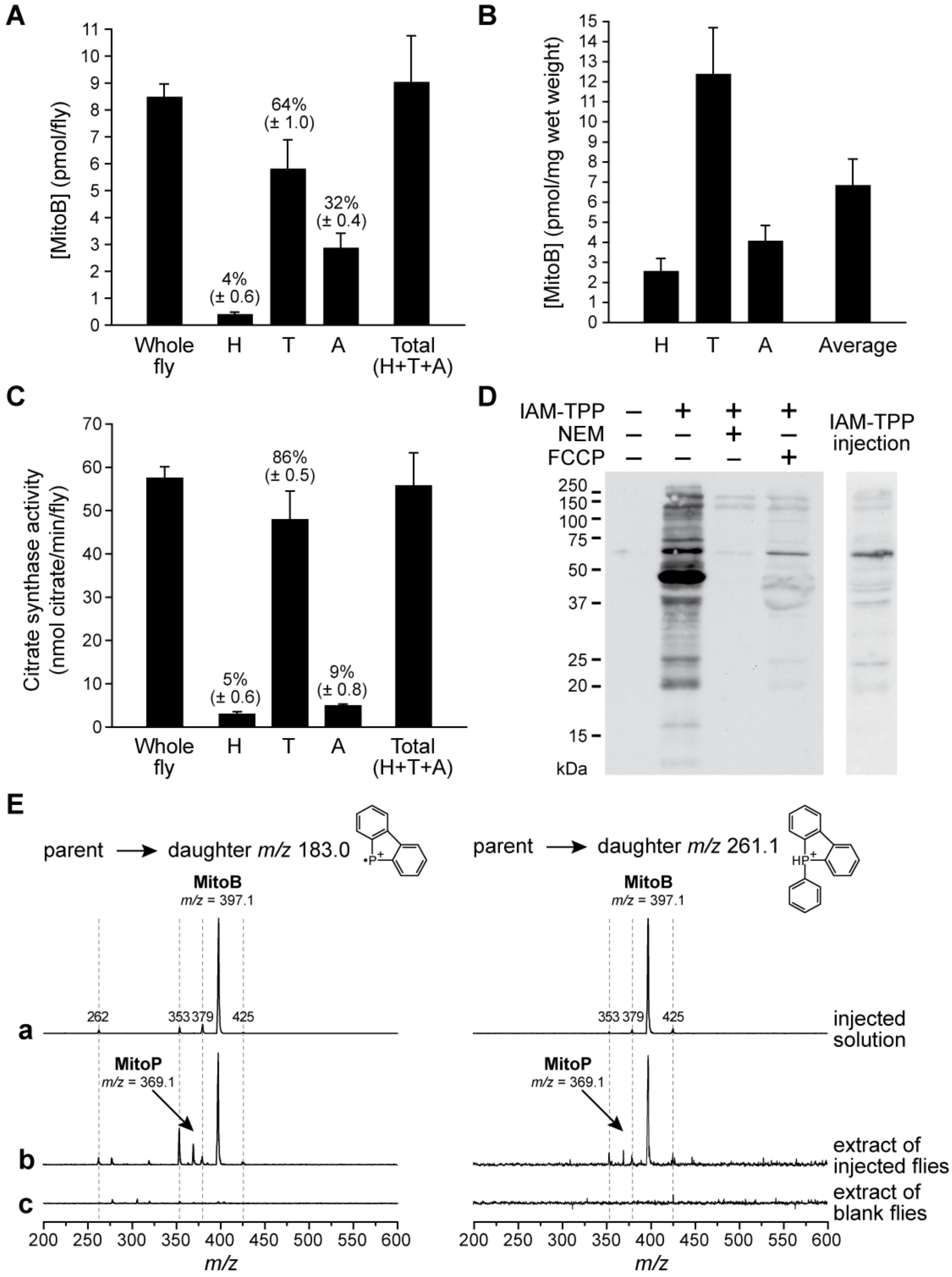
A
[MitoB] (pmol/fly)
4%
(± 0.6)
64%
(± 1.0)
32%
(± 0.4)
Whole fly
H
T
A
Total (H+T+A)
B
[MitoB] (pmol/mg wet weight)
H
T
A
Average
C
Citrate synthase activity (nmol citrate/min/fly)
5%
(± 0.6)
86%
(± 0.5)
9%
(± 0.8)
Whole fly
H
T
A
Total (H+T+A)
D
IAM-TPP
NEM
FCCP
IAM-TPP injection
250
150
100
75
50
37
25
20
15
kDa
E
parent → daughter m/z 183.0
parent → daughter m/z 261.1
MitoB
m/z = 397.1
MitoP
m/z = 369.1
262
353
379
425
a
b
c
injected solution
extract of injected flies
extract of blank flies
m/z

Figure S5. Uptake and Metabolism of MitoB by Mitochondria Within Flies

(A) Distribution of MitoB within flies. Wild-type females (7 d) were injected with MitoB and incubated for 3 h. Following snap freezing, the flies (cohorts of 20) were dissected into body parts, and the amount of MitoB in the homogenates from head (H), thorax (T) and abdomen (A) was measured by LC/MS/MS. Data are means ± SEM of three determinations. The % of total MitoB in each body part relative to the sum in all is indicated (mean ± SD, n = 3).
(B) Concentration of MitoB within females flies. Data from (A) were divided by the wet weight of the individual body parts. The average MitoB concentration was determined by dividing the total amount of MitoB in the three body parts by the sum of the wet weight of the three body parts. Data are means ± SEM of three determinations.
(C) Distribution of mitochondria within wild-type female flies (7 d). Total citrate synthase activity was measured in homogenates prepared from whole flies, or from flies dissected into body parts (cohorts of 10). Data are means ± SEM of three separate measurements. The % of total citrate synthase activity in each body part expressed relative to the sum in all is indicated (mean ± SD, n = 3). The specific activities for citrate synthase in the whole body, head, thorax and abdomen respectively were: 39 ± 7; 38 ± 10; 84 ± 14; 7 ± 1 nmol citrate/min/mg protein (means ± SEM, n = 3).
(D) Western blots showing mitochondrial labelling with iodoacetamide-TPP (IAM-TPP).
Left panel - control mitochondria (50 μg protein) were energized with glycerol-3-phosphate (10 mM) and incubated at 25ºC for 15 min with IAM-TPP (5 μM). This resulted in TPP-labelling of mitochondrial proteins on cysteine residues that was visualized using antiserum against TPP. Blocking cysteine thiols with *N*-ethylmaleimide (NEM, 100 μM) prevented IAM-TPP binding. Uncoupling with FCCP (1 μM) prevented the $\Delta\psi_m$-dependent accumulation of IAM-TPP inside mitochondria and thereby decreased labelling of proteins. Right panel - live flies (~150 7 d females) were injected with 75 pmol IAM-TPP per fly in the same way as was done for MitoB. After incubating the injected flies for 3 h, mitochondria were isolated and 50 μg mitochondrial protein was assessed for TPP labelling. The banding pattern obtained for TPP labelling of the mitochondria from flies that had been injected with IAM-TPP was very similar to that of control mitochondria that were incubated with IAM-TPP *in vitro*. These findings confirm the mitochondrial localization of IAM-TPP *in vivo*. IAM-TPP labelling was not detectable in cytosolic fractions from injected flies (data not shown).
(E) Metabolites of MitoB Within Flies Assessed by Tandem Mass Spectrometry. Ten cohorts of 10 flies were injected with MitoB, incubated for 6 h and then homogenized and extracted without internal standards. The extracts were dried, then resuspended and combined in a total volume of 200 μl 40% acetonitrile/0.1% formic acid. Samples of the MitoB solution that was injected into the flies (traces a), extracts from the MitoB injected flies (traces b) and extracts of a control cohort of uninjected flies (traces c) were analyzed for molecules that contained the TPP moiety. To do this the samples were directly infused into the mass spectrometer at 5

µl/min for 1 min, and the *m/z* of parent ions that fragmented to give a daughter ion of *m/z* = 183.0 (left), or of *m/z* = 261.1 (right), both diagnostic of the TPP moiety, were identified. For traces A and B each trace is normalized to the highest total ion count peak within that trace, hence baseline noise is magnified in the traces for the daughter ion of *m/z* = 261.1 relative to that for the daughter ions of *m/z* = 183.0 as the total ion count is ~10-fold higher in the latter. The background traces (c) for the extracts from uninjected flies are normalized to the highest total ion count peak within the corresponding trace (b) to equalize baseline noise and to facilitate comparison. The prominent parent ion of MitoB (*m/z* 397.1) and a few contaminants present in the injectate were all detectable in the injected flies (dashed lines). The only new peak present in the injected flies that was not present in the injectate was at *m/z* = 369.1 corresponding to MitoP. Therefore within flies MitoB is only metabolized to MitoP.

Figure S6

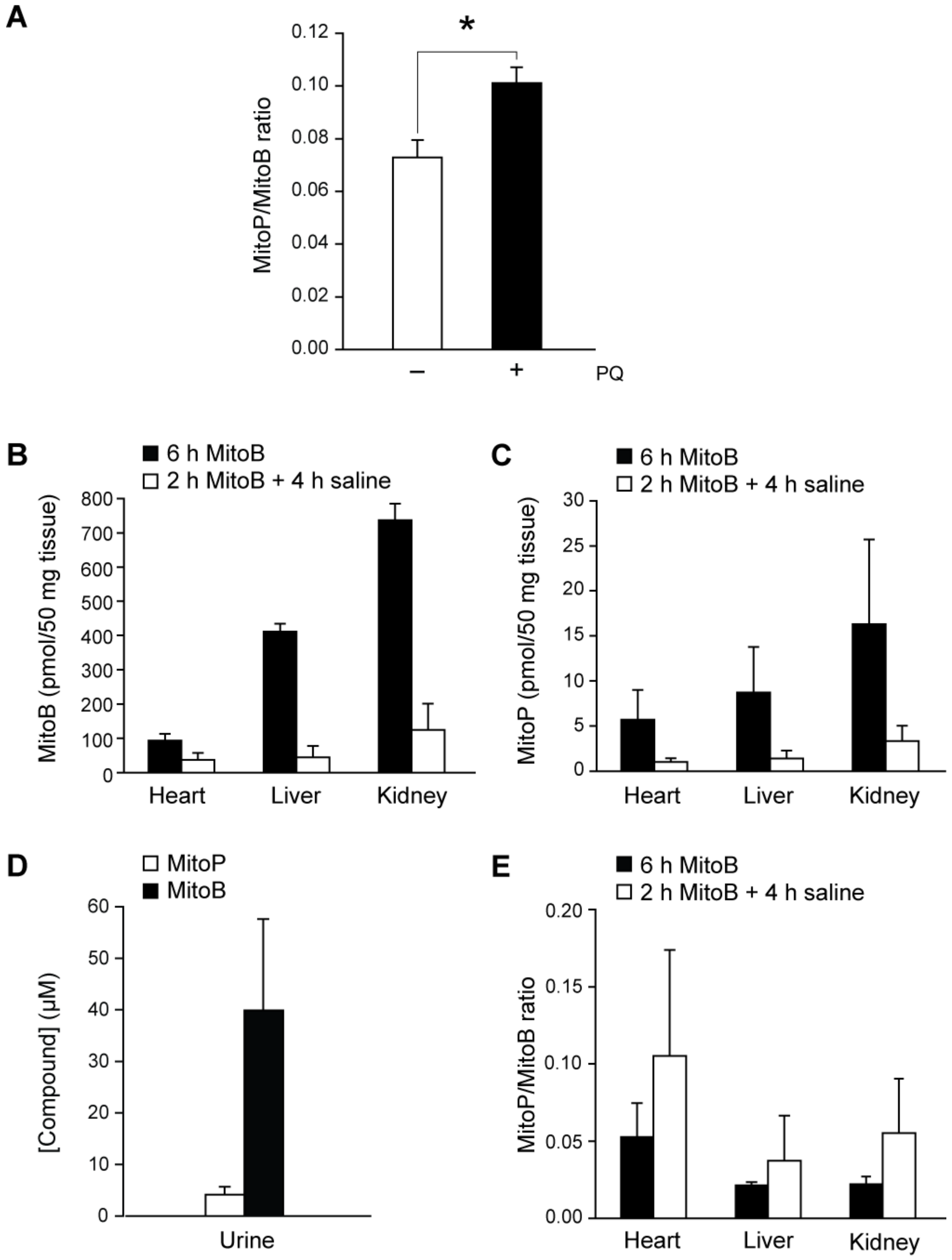

A
*
0.12
0.10
0.08
0.06
0.04
0.02
0.00
MitoP/MitoB ratio
–
+
PQ
B
6 h MitoB
2 h MitoB + 4 h saline
MitoB (pmol/50 mg tissue)
800
700
600
500
400
300
200
100
0
Heart
Liver
Kidney
C
6 h MitoB
2 h MitoB + 4 h saline
MitoP (pmol/50 mg tissue)
30
25
20
15
10
5
0
Heart
Liver
Kidney
D
MitoP
MitoB
[Compound] (μM)
60
50
40
30
20
10
0
Urine
E
6 h MitoB
2 h MitoB + 4 h saline
MitoP/MitoB ratio
0.20
0.15
0.10
0.05
0.00
Heart
Liver
Kidney

Figure S6. Using MitoB to Assess Mitochondrial ROS Production in *C. elegans* and Mice

(A) Effect of paraquat on the MitoP/MitoB ratio within worms. Wild-type *C. elegans* (N2 strain) were incubated for 1 h with 10 μM MitoB and 50 U catalase ± 50 mM paraquat (PQ), then the MitoP/MitoB ratio in the worm pellet was quantified by LC/MS/MS. Data are means ± SEM of four samples, and were corrected for the ratio at t = 0. There was also a statistically significant increase in the MitoP/MitoB ratio in the worm incubation medium following treatment with 100 mM paraquat (data not shown). Statistical significance was determined by a two-tailed Student's *t*-test: * $p < 0.05$.

(B,C) To see if the MitoP/MitoB ratio could be measured within mammalian tissues, mice were infused intravenously with 180 nmol of MitoB over 6 h, or infused with 180 nmol of MitoB over 2 h followed by 4 h saline infusion with no MitoB. The mice were then sacrificed and the MitoB and MitoP content of tissue samples was assessed by extraction followed by LC/MS/MS analysis relative to deuterated standards. Data are means ± SEM of three-four mice for each condition. After 6 h MitoB infusion, the amount of MitoB in the blood was 22 ± 7 pmol MitoB/50 mg blood (n = 3, mean ± SEM), far lower that the amount present within tissues. By 4 h after the 2 h MitoB infusion, there was no MitoB detectable in the blood, despite large amounts in the tissues. These findings are consistent with the rapid uptake of MitoB from the circulation into tissues, as has been found for other TPP cations (Porteous, 2010; Smith et al., 2003).

(D) Mice were administered with MitoB (180 nmol of MitoB over 6 h) and the levels of MitoB and MitoP in the urine were measured. Data are means ± range for two mice.

(E) Ratio of MitoP/MitoB in mouse tissues. Mice were administered MitoB and the ratios of MitoP/MitoB in the tissues were determined after MitoB infusion for 6 h or after MitoB infusion for 2 h followed by 4 h saline infusion with no MitoB. Data are means ± SEM of three-four mice for each condition. The MitoP/MitoB ratio in the tissues showed that the 4 h period post-infusion resulted in a 1.6 to 2.5-fold increase in the MitoP/MitoB ratio relative to continuous infusion, consistent with conversion of MitoB to MitoP by mitochondrial ROS *in vivo*.

Figure S7

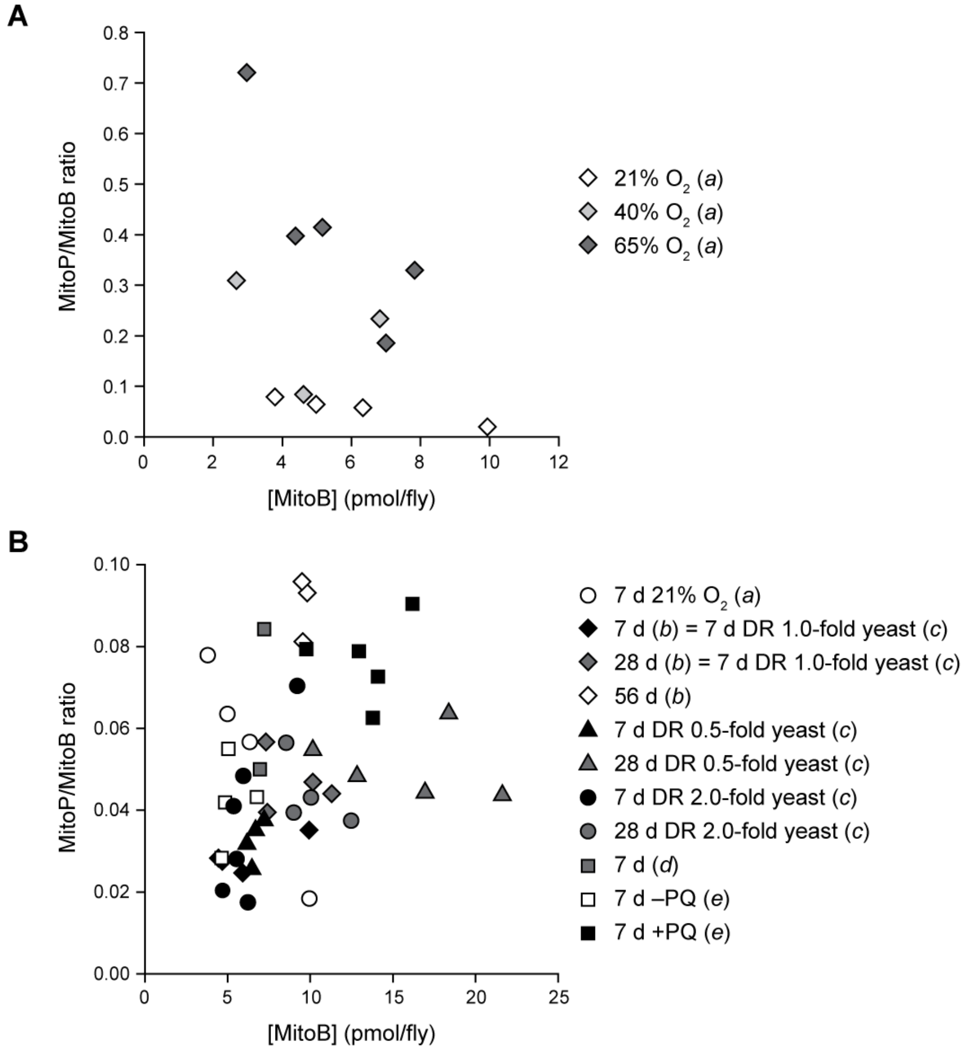

Figure S7. Lack of Correlation Between the Absolute Amount of MitoB Expressed in pmol per Fly and the Corresponding MitoP/MitoB Ratio Obtained

Each point represents an individual cohort of 10 flies in which the MitoB content and the MitoP/MitoB ratio was measured. Letters in italics denote separate experiments.

(A) Effect of hyperoxia. All data are for wild-type 7 d female flies incubated for 6 h after injection with MitoB.

(B) Effect of aging, dietary restriction (DR) and paraquat (PQ) treatment. All data are for wild-type female flies incubated for 6 h after injection with MitoB.

SUPPLEMENTAL CALCULATIONS

Calculation of the Mitochondrial Content of MitoB *In Vivo* Within Living Flies

To estimate the amount of MitoB present within flies that was inside mitochondria, we first estimated the proportion of the MitoB that was present within cells inside the flies (α). To do this, we consider the known content and distribution of water within flies: female adult flies, which weigh approximately 1.5 mg, contain about 66% by weight total body water of which about 6-10% is hemolymph with the remaining 90-94% being cellular water (Folk et al., 2001). Thus, we take the proportion of MitoB within the fly that is intracellular, α, as 0.9. This is likely to be an underestimate as MitoB in tissues is concentrated into the cells driven by the plasma membrane potential ($\Delta\psi_p$) with subsequent uptake into the mitochondria driven by the mitochondrial membrane potential ($\Delta\psi_m$). This is consistent with the findings in mice administered MitoB (this paper) and other TPP compounds (Porteous et al., 2010) in which all TPP compounds were rapidly removed from the circulation and concentrated within the tissues.

The next factor to be determined is the proportion of intracellular MitoB that is present within the mitochondria (β). This value depends on the concentration of MitoB in the mitochondrial and cytoplasmic compartments ($[MitoB]_{mito}$ and $[MitoB]_{cyto}$, respectively), and the volumes of those compartments (Vol_{mito} and Vol_{cyto}, respectively), and is given by:

(1) $$\beta = \frac{Vol_{mito}[MitoB]_{mito}}{Vol_{cyto}[MitoB]_{cyto} + Vol_{mito}[MitoB]_{mito}}$$

This can be arranged to:

(2) $$\beta = \frac{\frac{Vol_{mito}[MitoB]_{mito}}{Vol_{cyto}[MitoB]_{cyto}}}{1 + \frac{Vol_{mito}[MitoB]_{mito}}{Vol_{cyto}[MitoB]_{cyto}}}$$

The ratio of MitoB concentrations in the mitochondrial and cytosolic compartments can be calculated from the mitochondrial membrane potential ($\Delta\psi_m$) using the Nernst equation, allowing for binding corrections for MitoB in the mitochondrial (a_{mito}) and cytosolic (a_{cyto}) compartments (Brand, 1995). The distribution of MitoB between the cytosol and mitochondria is then determined by the $\Delta\psi_m$ which in mV at 25ºC is:

(3) $$\Delta\psi_m = 59.5\log_{10}\frac{a_{mito}[MitoB]_{mito}}{a_{cyto}[MitoB]_{cyto}}$$

This can be rearranged to give the ratio of the concentration of MitoB in the two compartments:

(4) $$\frac{[MitoB]_{mito}}{[MitoB]_{cyto}} = \frac{a_{cyto}}{a_{mito}}10^{\frac{\Delta\psi m}{59.5}}$$

Therefore the proportion of intracellular MitoB that is within the mitochondria, β, is given by:

(5) $$\beta = \frac{\frac{Vol_{mito}a_{cyto}}{Vol_{cyto}a_{mito}}10^{\frac{\Delta\psi m}{59.5}}}{1+\frac{Vol_{mito}a_{cyto}}{Vol_{cyto}a_{mito}}10^{\frac{\Delta\psi m}{59.5}}}$$

The volume fraction of the *Drosophila* flight muscle that is mitochondrial has been determined by stereology of electron micrographs as 35-40% (Magwere et al., 2006). The average mitochondrial density within whole male flies has been estimated as ~30% from measuring the ratio of the specific activities of four mitochondrial enzymes in isolated mitochondria to their activities in whole fly homogenates (Magwere et al., 2006). Therefore it is reasonable to take the average mitochondrial volume within cells inside flies as 30% so Vol_{mito}/Vol_{cyto} = 0.428. The mitochondrial binding correction factor for MitoB (a_{mito}) can be assumed to be the same as that of the closely related molecule methylTPP calculated for fly mitochondria: a_{mito} = 0.24 (Brand et al., 2005). The cytosolic binding correction factor for MitoB (a_{cyto}) can be assumed to be the same as for

methylTPP in the cytosol of hepatocytes: a_{cyto} = 0.21 (Brand, 1995). Therefore a_{cyto}/a_{mito} = 0.875. Entering these values into equation (5) gives:

$$\beta = \frac{0.375 \times 10^{\frac{\Delta\psi_m}{59.5}}}{1 + 0.375 \times 10^{\frac{\Delta\psi_m}{59.5}}} \quad (6)$$

If we take a reasonable value for the average $\Delta\psi_m$ within flies of 140 mV, then β = 0.98. If we vary the $\Delta\psi_m$ to 120 or 160 mV, then β ranges between 0.97 and 0.99 respectively. Therefore we take the fraction of the intracellular MitoB that is mitochondrial (β) as 0.98. Multiplying β by the fraction of MitoB within flies that is present within cells, α, which is 0.9 giving the fraction of total MitoB in the fly that is in the mitochondria: α x β = 0.98 x 0.9 = 0.88.

Calculation of Average Mitochondrial [H_2O_2] *In Vivo* Within Living Flies

To calculate the average concentration of H_2O_2 within mitochondria in living flies, $[H_2O_2]_{mito}$, from the measured MitoP/MitoB ratio requires two assumptions. The first is that within the fly the only significant place where MitoB reacts with H_2O_2 to form MitoP is within mitochondria. This is justified because, as indicated above, most (~88%) of the MitoB within the fly is present within mitochondria. As the reaction of MitoB with H_2O_2 is second order, the several hundred-fold concentration of MitoB within mitochondria will lead to a similar fold enhancement of its reaction rate with mitochondrial H_2O_2. In addition, the pH within the mitochondrial matrix (~8.0) will increase the reactivity of MitoB with H_2O_2 by about ~4-fold relative to that in the cytosol (pH 7.2). The second assumption is that the MitoP/MitoB ratio equilibrates rapidly throughout the fly compared to the timescale of ~3-6 h over which the MitoB experiments are carried out. This assumption is reasonable as the conversion of MitoB to MitoP by H_2O_2 is slow (3.8 $M^{-1}s^{-1}$ at 25ºC, pH 8.0), while TPP cations are known to equilibrate between different compartments relatively rapidly over minutes within cells (Ross et al., 2008) and mice (Porteous et al., 2010), and is further supported by the rapid uptake and release of MitoB and MitoP by

mitochondria and cells, and the distribution of MitoB throughout the body of the fly following injection.

From these assumptions, and from the findings reported here, the total MitoB concentration within the fly ($[MitoB]_{total}$) is eliminated from the body (*E*) by a first order reaction with a rate constant k_1. MitoP is only generated within the mitochondria by reaction of $[MitoB]_{mito}$ with $[H_2O_2]_{mito}$ by a second order reaction with constant k_2. $[MitoB]_{total}$ can be related to $[MitoB]_{mito}$, by the factor γ: $[MitoB]_{mito} = \gamma[MitoB]_{total}$. Finally, once formed, MitoP within mitochondria equilibrates with the whole body $[MitoP]_{total}$ which is then eliminated from the body (*E*) by a first order process with a rate constant k_3. Together this leads to a relationship between the measureable quantities, $[MitoB]_{total}$ and $[MitoP]_{total}$ within the fly, and $[H_2O_2]_{mito}$:

(7) $$E \xleftarrow{k_1[MitoB]_{total}} [MitoB]_{total} \xrightarrow{k_2[H_2O_2]_{mito}(\gamma[MitoB]_{total})} [MitoP]_{total} \xrightarrow{k_3[MitoP]_{total}} E$$

From equation 7 we can derive the relevant rate equation for the change in $[MitoB]_{total}$:

(8) $$\frac{d[MitoB]_{total}}{dt} = -(k_1 + \gamma k_2[H_2O_2]_{mito})[MitoB]_{total}$$

Solving (8) gives:

(9) $$[MitoB]_{total} = [MitoB(t=0)]_{total} e^{-(k_1+\gamma k_2[H_2O_2]mito)t}$$

From equations 7 and 9 we can derive the relevant rate equations for the change in $[MitoP]_{total}$:

(10) $$\frac{d[MitoP]_{total}}{dt} = \gamma k_2[H_2O_2]_{mito}[MitoB(t=0)]_{total} e^{-(k_1+\gamma k_2[H_2O_2]mito)t} - k_3[MitoP]_{total}$$

Solving equation 10 for $[MitoP]_{total}$ gives:

(11) $$[MitoP]_{total} = \frac{\gamma k_2[H_2O_2]_{mito}}{k_3 - k_1 - \gamma k_2[H_2O_2]_{mito}}(e^{-(k_1+\gamma k_2[H_2O_2]mito)t} - e^{-k_3 t})[MitoB(t=0)]_{total}$$

As the rates of elimination of MitoB and MitoP from the whole fly are the same (Figure 5A) $k_1 = k_3$. Consequently the equation then simplifies to:

(12) $$[MitoP]_{total} = (e^{k_1 t} - e^{-(k_1 + \gamma k_2 [H_2O_2]_{mito})t})[MitoB(t=0)]_{total}$$

Dividing equation 12 by equation 9 gives the experimentally measured MitoP/MitoB ratio:

(13) $$\frac{[MitoP]_{total}}{[MitoB]_{total}} = (e^{(\gamma k_2 [H_2O_2]_{mito})t} - 1)$$

Which can be rearranged to give $[H_2O_2]_{mito}$:

(14) $$[H_2O_2]_{mito} = \frac{1}{\gamma k_2 t} \log_e \left(\frac{[MitoP]_{total}}{[MitoB]_{total}} + 1\right)$$

Therefore it is possible to calculate the average $[H_2O_2]_{mito}$ from the experimentally accessible MitoP/MitoB ratio and γ, the ratio of $[MitoB]_{mito}/[MitoB]_{total}$, which can be derived from experimentally measureable factors, with a minimal number of assumptions.

Calculation of $[MitoB]_{mito}/[MitoB]_{total}$ Within Flies

Equation 14 gives $[H_2O_2]_{mito}$, but requires knowledge of $\gamma = [MitoB]_{mito}/[MitoB]_{total}$. To estimate γ in terms of measureables, we can take alternative expressions for $[MitoB]_{mito}$ and $[MitoB]_{total}$, based on the moles of MitoB present in the mitochondria and within the whole fly *in vivo*, $Mol(MitoB)_{mito}$ and $Mol(MitoB)_{total}$, and the volume of the MitoB accessible mitochondrial and whole body compartments, Vol_{mito} and Vol_{total}.

(15) $$[MitoB]_{mito} = \frac{Mol(MitoB)_{mito}}{Vol_{mito}}$$

(16) $$[MitoB]_{total} = \frac{Mol(MitoB)_{total}}{Vol_{total}}$$

Rearranging gives:

(17) $$\gamma = \frac{Mol(MitoB)_{mito}}{Mol(MitoB)_{total}} \bullet \frac{Vol_{total}}{Vol_{mito}}$$

$Mol(MitoB)_{mito}/Mol(MitoB)_{total}$ is the same as the fraction of total MitoB in the fly that is mitochondrial (= α x β) which we earlier estimated to be 0.88. Vol_{total}/Vol_{mito} is the reciprocal of the product of the proportion of the total fly body water that is cellular (= 0.90 (Folk et al., 2001)) by the proportion of the cell that is mitochondrial, which is 0.30 (Magwere et al., 2006). Together these give γ= 3.26.

SUPPLEMENTAL EXPERIMENTAL PROCEDURES

Synthesis of MitoB and MitoP

All reactions under an inert atmosphere were carried out using oven-dried or flame-dried glassware. Solutions were added by syringe. Acetonitrile, tetrahydrofuran and toluene were dried where necessary using a solvent drying system, Puresolv™. Reagents were obtained from commercial suppliers and used without further purification unless otherwise stated. ^{1}H, ^{31}P and ^{13}C NMR spectra were obtained on a Bruker DPX/400 spectrometer operating at 400, 162 and 100 MHz respectively. All coupling constants are measured in Hz. ^{31}P and ^{13}C NMR spectra are proton-decoupled. DEPT (distortionless enhancement by polarization transfer) was used to assign the signals in the ^{13}C NMR spectra as C, CH, CH_2 or CH_3. Signals for deuterated carbons of perdeuterated aromatic rings appear as narrow multiplets and are given the assignment CD. ESI-MS were carried out on a Thermofisher LTQ Orbitrap XL at the University of Swansea, other mass spectra (MS) were recorded on a Jeol JMS700 (Mstation) spectrometer. Infrared (IR) spectra of compounds in either solid or liquid form were obtained on a Shimadzu FTIR-8400S using attenuated total reflectance (ATR). Microanalyses were obtained on an Exeter Analytical CE440. This instrument cannot distinguish between hydrogen and deuterium and the percentages of D and H found in deuterated compounds are calculated from raw data with any deviation from theoretical appearing in the percentage D.

2-(3'-Methylphenyl)-4,4,5,5-tetramethyl-1,3,2-dioxaborolane **2***.* ^{t}BuLi (21.0 ml of a 1.7 M solution in pentane, 35.1 mmol) was added drop-wise to a stirred solution of aryl bromide **1** (3.00 g, 17.5 mmol) in dry tetrahydrofuran (40 ml) under argon at –78ºC. After 15 min, 2-isopropoxy-4,4,5,5-tetramethyl-1,3,2-dioxaborolane (11.9 ml, 58.3 mmol) was added drop-wise. The solution was allowed to stir for 1 h before quenching with ice-cold H_2O (20 ml). The mixture was extracted with ethyl acetate (EtOAc; 30 ml) and the organic layer washed with brine (30 ml), dried over $MgSO_4$ and concentrated *in vacuo* at 60ºC for 3 h affording the arylboronate **2** as an oil (3.59 g, 94%). δ_H ($CDCl_3$, 400 MHz): 7.64-7.60 (2H, m, ArH), 7.28-7.27 (2H, m, ArH),

2.36 (3H, s, CH_3Ar), 1.35 (12H, 2 × Me_2C). δ_C ($CDCl_3$, 100 MHz): 137.18 (C), 135.50 (CH), 132.18 (CH), 131.96 (CH), 127.84 (CH), 83.80 (C), 25.00 (CH_3), 21.42 (CH_3). IR (ATR, cm^{-1}): 2982 (C-H), 2924 (C-H), 1605 (Ar), 1582 (Ar). MS, (CI^+), *m/z*: 219 ($M+H^+$, 100%). HRMS: 219.1553 ($M+H^+$). $C_{13}H_{20}O_2{}^{11}B$ requires 219.1556. ^1H and ^{13}C NMR in good agreement with literature (Ishiyama et al., 2002).

2-[3'-(Bromomethyl)phenyl]-4,4,5,5-tetramethyl-1,3,2-dioxaborolane **3.** *N*-bromosuccinimide (1.71 g, 9.6 mmol) and benzoyl peroxide (40 mg, 0.2 mmol) were added to a stirred solution of the arylboronate **2** (2.00 g, 9.2 mmol) in CCl_4 (25 ml). The resulting mixture was heated under reflux overnight. The mixture was allowed to cool and then concentrated *in vacuo*. The resulting oil was dissolved in DCM (100 ml) and washed with H_2O (100 ml). The organic layer was dried over $MgSO_4$, concentrated to dryness and the resulting yellow solid was recrystallized from hexane affording the bromide **3** as prisms (1.47 g, 54%). Mp: 89-92°C. δ_H ($CDCl_3$, 400 MHz): 7.82 (1H, s, H-2'), 7.74 (1H, d, *J* = 7.5 Hz, H-6'), 7.50 (1H, d, *J* = 7.5 Hz, H-4'), 7.36 (1H, t, *J* = 7.5 Hz, H-5'), 4.50 (2H, s, CH_2Br), 1.35 (12H, s, 2 × Me_2C). δ_C ($CDCl_3$, 100 MHz): 137.24 (C), 135.32 (CH), 134.92 (CH), 132.10 (CH), 128.40 (CH), 84.09 (C), 33.63 (CH_2), 25.00 (CH_3). IR (ATR, cm^{-1}): 2978 (C-H), 1602 (Ar). MS, (CI^+), *m/z*: 299 [$M+H^+$ (^{81}Br), 26%], 297 [$M+H^+$ (^{79}Br), 26], 219 (53), 217 (57), 89 (100). HRMS: 297.0665 [$M+H^+$ (^{79}Br)] and 299.0634 [$M+H^+$ (^{81}Br)]. $C_{13}H_{19}O_2{}^{79}Br^{11}B$ requires 297.0662 and $C_{13}H_{19}O_2{}^{81}Br^{11}B$ requires 299.0641. This compound was mentioned in literature (Howard et al., 2001), but no spectral data were provided.

[3-(4',4',5',5'-Tetramethyl-1',3',2'-dioxaborolan-2'-yl)benzyl]triphenylphosphonium bromide, Mito B pinacol ester. A solution of benzylic bromide **3** (500 mg, 1.7 mmol) and triphenylphosphine (446 mg, 1.7 mmol) were heated under reflux in dry toluene (5 ml) under argon for 36 h. The precipitate formed was filtered off and recrystallized from EtOAc-hexane yielding Mito B pinacol ester as an amorphous solid (360 mg, 38%). Mp: > 220ºC (Decomp.). δ_H (CD_3OD, 400 MHz): 7.93-7.90 (3H, m, 3 × *p*-H of PPh_3), 7.76-7.63 (13H, m, H-6 and 6 × *o*-H and 6 × *m*-H of PPh_3), 7.31 (1H, s, H-2), 7.27 (1H, t, *J* = 7.6 Hz, H-5), 7.17 (1H, d, *J* = 7.6 Hz, H-4), 4.92 (2H, d, *J* = 14.9 Hz, CH_2P), 1.30 (12H, s, 2 × Me_2C). δ_C (CD_3OD,

100 MHz): 138.71 (CH), 136.46 (CH, d, *J* =3.0 Hz), 135.85 (CH), 135.41 (CH, d, *J* = 9.7 Hz), 134.91 (CH, *J* = 5.6 Hz), 131.34 (CH, d, *J* = 12.2 Hz), 129.54 (CH), 128.09 (C, d, *J* = 8.1 Hz), 119.06 (C, d, *J* = 85.7 Hz), 85.26 (C), 30.72 (CH_2, *J* = 48.5 Hz), 25.18 (CH_3). IR (ATR, cm^{-1}): 2970 (C-H), 2859 (C-H), 2839 (C-H), 2778 (C-H), 1435 (PPh_3). LRMS (FAB/NOBA): 479 [M^+ (phosphonium cation), 100%]. HRMS: 479.2307 [M^+ (phosphonium cation)]. $C_{31}H_{33}O_2P^{11}B$ requires 479.2308.

[3-(4',4',5',5'-Tetramethyl-1',3',2'-dioxaborolan-2'-yl)benzyl]-tris(pentadeuterophenyl) phosphonium bromide, *d₁₅*-MitoB pinacol ester. A solution of benzylic bromide **3** (500 mg, 1.7 mmol) and d_{15}-triphenylphosphine (446 mg, 1.7 mmol) were heated under reflux in dry toluene (3 ml) under argon for 24 h. The precipitate formed was filtered off and then recrystallized from EtOAc-hexane yielding d_{15}-MitoB pinacol ester as an amorphous solid (790 mg, 81%). Mp: > 220°C (Decomp.). δ_H (CD_3OD, 400 MHz): 7.69 (1H, d, *J* = 7.6 Hz, H-6), 7.29 (1H, s, H-2), 7.26 (1H, t, *J* = 7.6 Hz, H-5), 7.16 (1H, d, *J* = 7.6 Hz, H-4), 4.75 (2H, d, *J* = 15.2 Hz, CH_2P), 1.29 (12H, s, 2 × Me_2C). δ_C (CD_3OD, 100 MHz): 138.78 (CH, *J* = 5.0 Hz), 135.79 (CH, *J* = 3.1 Hz), 136.20-134.55 (m, CD), 131.09-130.71 (m, CD), 129.54 (CH, *J* = 3.2 Hz), 128.18 (C, d, *J* = 9.1 Hz), 117.51 (C, d, *J* = 86.6 Hz), 85.31 (C), 30.81 (CH_2, *J* = 47.3 Hz), 25.25 (CH_3). IR (ATR, cm^{-1}): 2974 (C-H), 2868 (CH), 2839 (C-H), 2777 (C-H), 1433 (PPh_3). LRMS (FAB/NOBA): 494 [M^+ (phosphonium cation), 100%]. HRMS: 494.3247 [M^+ (phosphonium cation)]. $C_{31}H_{33}O_2P^{11}B$ requires 494.3247.

(3-Hydroxybenzyl)triphenylphosphonium bromide, MitoP. Method A (original method). Aqueous hydrogen peroxide (1.7 ml, 50% w/w, 0.84 mmol) was added to a stirred solution of the benzylic bromide **3** (50 mg, 0.17 mmol) in a 1:2 mixture of saturated aqueous $NaHCO_3$ and MeOH (5 ml). The resulting mixture was stirred for 2 h then diluted with H_2O (5 ml) and extracted into Et_2O (5 ml). The organic layer was dried over $MgSO_4$ and concentrated *in vacuo* to afford the phenol as an oil. Due to the instability of the phenol, it was carried on without further purification. The bromide was dissolved in toluene (3 ml) and triphenylphosphine (45 mg, 0.17 mmol) added. The reaction mixture was stirred and heated under reflux overnight. The precipitate formed was filtered off and recrystallized from EtOH to give (3-hydroxybenzyl)triphenylphosphonium bromide (53 mg, 56%) as an amorphous solid.

Mp: > 290ºC (Decomp.). δ_H (400 MHz, d_6–DMSO): 9.54 (1H, s, OH), 7.94-7.86 (3H, m, 3 × *p*-H PPh_3), 7.78-7.70 (6H, m, 6 × *m*-H PPh_3), 7.70-7.60 (6H, m, 6 × *o*-H PPh_3), 7.00 (1H, t, *J* = 7.8 Hz, H-5), 6.68 (1H, broad d, *J* = 8.0 Hz, H-6), 6.41 (1H, broad s, H-2), 6.36 (1H, broad d, *J* = 7.1 Hz H-4), 5.06 (2H, d, *J* = 15.6 Hz, CH_2). δ_C (100 MHz, d_6–DMSO): 157.54 (d, *J* = 3.2 Hz, C), 135.10 (d, *J* = 1.9 Hz, CH), 134.03 (d, *J* = 9.8 Hz, CH), 130.11 (d, *J* = 12.5 Hz, CH), 129.75 (d, *J* = 2.2 Hz, CH), 129.05 (d, *J* = 8.3 Hz, C), 121.39 (d, *J* = 5.5 Hz, CH), 117.98 (d, *J* = 85.6 Hz, C), 117.83 (d, *J* = 5.6 Hz, CH), 115.33 (d, *J* = 3.1 Hz, CH), 28.13 (d, *J* = 46.6 Hz, CH_2). δ_P (162 MHz, d_6–DMSO): 23.03 (s). IR (ATR, cm^{-1}): 3079 (OH), 2889 (ArH), 2858 (ArH), 2787 (ArH), 1611 (Ar), 1568 (Ar). LRMS (ESI): 369 [M^+ (phosphonium cation), 100%]. HRMS: 369.1399 [M^+ (phosphonium cation)]. $C_{25}H_{22}OP$ requires 369.1403. Microanalysis: $C_{25}H_{22}BrOP$ requires C: 66.83% H: 4.94%, found C: 66.89% H: 4.98%. ^1H NMR data in broad agreement with those reported for the compound in $CDCl_3$ (Dawson et al., 1980).

(3-Hydroxybenzyl)triphenylphosphonium bromide, MitoP. Method B (recommended method for future work). Adapting the literature method (Dawson et al., 1980), PBr_3 (0.95 ml, 1.0 M in DCM, 3.50 mmol) and dry pyridine (150 µl, 1.85 mmol) were added to anhydrous acetonitrile (1 ml) at –8ºC, under argon and stirred for 5 min. A solution of 3-hydroxybenzyl alcohol **4** (1.00 g, 8.06 mmol) and dry pyridine (50 µl, 0.62 mmol) in dry acetonitrile (2.5 ml) was added slowly and the resulting solution stirred for 10 min at –8ºC, under argon. The solution was allowed to warm to RT and stirred at 20ºC for 15 h before being concentrated under reduced pressure. H_2O (10 ml) was added and extracted with DCM (2 × 10 ml). The extracts were eluted through a short silica column [EtOAc-DCM (1:19 to 1:9)]. Toluene (8 ml) was added to the crude yellow, unstable oil (1.13 g) followed by triphenylphosphine (3.58 g, 13.6 mmol) and the mixture stirred and heated under reflux for 16 h. The reaction was allowed to cool to RT and filtered. The precipitate was washed with hot toluene then Et_2O and dried under reduced pressure. The solid was recrystallized from EtOH yielding MitoP as prisms (2.31 g, 64%). Data as above.

(3-Hydroxybenzyl)tris(pentadeuterophenyl)phosphonium bromide, d_{15}-MitoP**.** d_{15}-MitoP was prepared in the same way as MitoP by method A, except using d_{15}-triphenylphosphine instead of triphenylphosphine. The benzylic bromide **3** (50 mg, 0.17

mmol) gave (3-hydroxybenzyl)tris(pentadeuterophenyl)phosphonium bromide (65 mg, 67%) as prisms after recrystallization from EtOH. Mp: > 290ºC (Decomp.). δ_H (400 MHz, d_6–DMSO): 9.55 (1H, s, OH), 7.00 (1H, t, J = 7.8 Hz, H-5), 6.69 (1H, broad d, J = 8.0 Hz, H-6), 6.42 (1H, broad s, H-2), 6.36 (1H, broad d, J = 7.2 Hz, H-4), 5.08 (2H, d, J = 15.7 Hz, CH_2). δ_C (100 MHz, d_6–DMSO): 157.54 (d, J = 3.1 Hz, C), 134.90-134.23 (m, CD), 134.08- 133.12 (m, CD), 130.08-129.20 (m, CD), 129.73 (d, J = 3.4 Hz, CH), 128.57 (d, J = 8.6 Hz, C), 121.39 (d, J = 5.6 Hz, CH), 117.85 (d, J = 5.5 Hz, CH), 117.76 (d, J = 85.4 Hz, C), 115.32 (d, J = 3.4 Hz, CH), 28.16 (d, J = 46.6 Hz, CH_2). δ_P (162 MHz, d_6–DMSO): 22.92 (s). IR (ATR, cm^{-1}): 3080 (OH), 2889 (ArH), 2858 (ArH), 2787 (ArH), 1617 (Ar), 1609 (Ar), 1587 (Ar). LRMS (ESI): 384 [M^+ (phosphonium cation), 100%]. HRMS: 384.2336 [M^+ (phosphonium cation)]. $C_{25}H_7D_{15}OP$ requires 384.2344. Microanalysis: $C_{25}H_7D_{15}BrOP$ requires C: 64.66% H: 1.52% D: 6.51%, found C: 64.69% H: 1.52% D: 6.69%.

3-(Dihydroxyboronyl)benzyltriphenylphosphonium bromide, MitoB**.** 3-(Bromomethyl)phenylboronic acid **5** (400 mg, 95%, 1.77 mmol) and triphenylphosphine (513 mg, 1.96 mmol) were heated under reflux in toluene (10 ml) for 6 h during which time a precipitate formed. The mixture was concentrated *in vacuo* and the residue was recrystallized from DMSO-toluene. The solid was dissolved in hot H_2O, and H_2O was removed *in vacuo* until precipitation began, upon which the mixture was allowed to cool to RT and to crystallize slowly. The mixture was centrifuged, and solvent removed by pipette. The resulting needles were washed with a little cold H_2O and dried *in vacuo* to yield the first batch of MitoB. A second batch of needles was obtained by repeating the recrystallization process with solid recovered from aqueous fractions by evaporation. The batches were combined to give MitoB (732 mg, 87%) as needles. Mp: 304-305ºC. δ_H (400 MHz, d_6–DMSO): 7.95 [2H, s, B(OH_2)], 7.93-7.86 (3H, m, PPh_3, 3 × p-H), 7.77-7.69 (7H, m, PPh_3, 6 × o-H and H-6), 7.68-7.60 (6H, m, PPh_3, 6 × m-H), 7.46 (1H, broad s, H-2), 7.18 (1H, t, J = 7.5 Hz, H-5), 6.94 (1H, broad d, J = 7.4 Hz, H-4), 5.12 (2H, d, J = 15.6 Hz, CH_2). δ_C (100 MHz, d_6–DMSO): 136.88 (d, J = 5.6 Hz, CH), 134.94 (d, J = 2.4 Hz, CH), 133.93 (d, J = 9.8 Hz, CH), 133.78 (d, J = 3.6 Hz, CH), 132.25 (d, J = 5.2 Hz, CH), 129.95 (d, J = 12.5 Hz, CH), 127.60 (d, J = 2.8 Hz, CH), 126.67 (d, J = 8.5 Hz, C), 117.75 (d, J = 85.5 Hz, C), 28.15 (d, J = 46.5 Hz, CH_2). δ_P (162 MHz, d_6–DMSO)

23.15 (s). IR (ATR, cm^{-1}): 3322 (OH), 2928 (ArH, v. weak), 2882 (ArH), 2789 (ArH, v. weak), 1549 (Ar, v. weak), 1485 (Ar, weak), 1435 (Ar, strong). LRMS (ESI): 397 [M^+ (phosphonium cation), 100%]. HRMS: 397.1518 [M^+ (phosphonium cation)]. $C_{25}H_{23}$ $^{11}BO_2P$ requires 397.1524. Microanalysis: $C_{25}H_{23}BBrO_2P$ requires C: 62.93% H: 4.86%, found C: 62.90% H: 4.80%.

[3-(Dihydroxyboronyl)benzyl]-tris(pentadeuterophenyl)phosphonium bromide d_{15}-MitoB**.** d_{15}-MitoB was prepared in the same way as MitoB except using d_{15}-triphenylphosphine instead of triphenylphosphine. 3-(Bromomethyl)phenylboronic acid **5** (155 mg, 95%, 0.69 mmol) gave d_{15}-MitoB (179 mg, 50%) as needles. Mp: 303-304ºC. δ_H (400 MHz, d_6–DMSO): 7.96 [2H, s, $B(OH)_2$], 7.72 (1H, broad d, J = 7.4 Hz, H-6), 7.46 (1H, broad s, H-2), 7.17 (1H, t, J = 7.6 Hz, H-5), 6.94 (1H, broad d, J = 7.6 Hz, H-4), 5.16 (2H, d, J = 15.6 Hz, CH_2). δ_C (100 MHz, d_6–DMSO): 137.02 (d, J = 5.6 Hz, CH), 135.08-134.22 (m, CD), 133.92 (d, J = 2.7 Hz, CH), 134.22-133.12 (m, CD), 132.43 (d, J = 5.5 Hz, CH), 130.05-129.15 (m, CD), 127.75 (d, J = 2.6 Hz, CH), 126.84 (d, J = 8.6 Hz, C), 117.67 (d, J = 85.4 Hz, C), 28.27 (d, J = 46.5 Hz, CH_2). δ_P (162 MHz, d_6–DMSO): 23.01 (s). IR (ATR, cm^{-1}): 3236 (OH), 2928 (ArH, v. weak), 2882 (ArH), 1543 (Ar), 1485 (Ar), 1424 (Ar, moderately strong). LRMS (ESI): 412 [M^+ (phosphonium cation), 100%]. HRMS: 412.2460 [M^+ (phosphonium cation)]. $C_{25}H_{23}{}^{11}BO_2P$ requires 412.2465. Microanalysis: $C_{25}H_8D_{15}BBrO_2P$ requires C: 61.00% H: 1.64% D: 6.14%, found C: 60.90% H: 1.64% D: 6.17%.

Characterization of MitoB and MitoP

The MitoB pinacol ester and d_{15}-MitoB pinacol ester were synthesized from 3-bromotoluene **1** (Figure S1A). The triphenylphosphine group is attached to a benzylic position in MitoB to allow easy synthesis and is *meta* to the boronate group, rather than *ortho* or *para*, so that fragmentation does not occur upon oxidation. Lithiation/boronation gave arylboronate **2** and radical bromination using *N*-bromosuccinimide then yielded benzylic bromide **3** (Howard et al., 2001). Displacement of the bromide by triphenylphosphine or d_{15}-triphenylphosphine gave MitoB pinacol ester and d_{15}-MitoB pinacol ester, respectively. Reaction between arylboronate **3** and basic hydrogen peroxide, followed by displacement of the

bromide from the resulting 3-(bromomethyl)phenol using triphenylphosphine or d_{15}-triphenylphosphine gave MitoP and d_{15}-MitoP, respectively. A better route to MitoP has been developed for future work adapting the method of Dawson et al. (Dawson et al., 1980) to prepare MitoP in two steps from 3-hydroxybenzyl alcohol **4** (Figure S1B). MitoB and d_{15}-MitoB were prepared by reaction between commercially available benzylic bromide **5** and triphenylphosphine or d_{15}-triphenylphosphine, respectively (Figure S1C).

Since Chang used the pinacol esters of boronic acids for the original H_2O_2-selective fluorescent probes (Dickinson and Chang, 2008; Miller et al., 2007), we first assessed the stability of MitoB pinacol ester by mass spectrometry. We found that the pinacol ester (m/z = 479.23) was very rapidly hydrolyzed to the arylboronic acid, MitoB itself (m/z = 397.15) in the 0.1% formic acid used for the LC/MS analysis, consistent with previous reports (Xu et al., 2006). As pinacol esters are also hydrolyzed by the silica matrix of the column during RP-HPLC (Xu et al., 2006) we analyzed the stability of the MitoB pinacol ester by direct infusion of solutions in 50% aqueous acetonitrile into the mass spectrometer. For freshly prepared stock solutions of MitoB pinacol ester in ethanol we could detect the pinacol ester but it decayed on storage so that all ethanolic stock solutions contained a significant amount of MitoB. Similarly, the MitoB pinacol ester was unstable on long-term storage in 50% aqueous acetonitrile. To assess stability of the pinacol ester during biological experiments, we incubated the MitoB pinacol ester in aqueous buffers at pH 7.2 and pH 8 and assessed stability by taking samples over time, making them up to 50% acetonitrile and directly infusing them into the mass spectrometer. This showed that MitoB pinacol ester was unstable in aqueous buffers with significant (~50%) decomposition to MitoB within 1 h at room temperature. This rapid hydrolysis in our experiments may in part have been due to the low concentrations of the pinacol esters used, consequently the dilution of the pinacol ester will drive the hydrolysis equilibrium to the right. This means that in most biological experiments even though the pinacol ester was added the active molecule is in fact the arylboronic acid. Consequently MitoB in the arylboronic acid form was used for all the biological experiments reported here to simplify analysis of its uptake and

distribution. It is likely that in Chang's work (Dickinson and Chang, 2008; Miller et al., 2007), the H_2O_2-selective fluorescent probes within cells are largely in the boronic acid form, although this does not affect the interpretation or significance of their experiments.

Absorbance spectra were performed in a UV-2501 PC spectrophotometer (Shimadzu). The local absorbance maxima in aqueous buffer were: MitoB, 268 nm (ε = 4,140 $M^{-1}cm^{-1}$) and 275 nm; MitoP, 275 nm (ε = 4,500 $M^{-1}cm^{-1}$) and 268 nm (ε = 4,420 $M^{-1}cm^{-1}$) with a shoulder at 290 nm (ε = 1,063 $M^{-1}cm^{-1}$). $\Delta\varepsilon_{MitoP - MitoB}$ at 285 nm = 1,744 $M^{-1}cm^{-1}$. The octan-1-ol/PBS partition coefficients were determined at 37ºC as described previously (Kelso et al., 2001): MitoB = 6.7 ± 0.9, MitoP = 3.3 ± 0.4 (means ± SD, n = 3). The rate constant obtained for the reaction of MitoB with H_2O_2 is consistent with rate constants reported for fluorescent H_2O_2 arylboronate probes (~0.2-1 $M^{-1}s^{-1}$ at pH 7.0, 25ºC (Dickinson and Chang, 2008; Miller et al., 2007)), 4-acetylphenylboronic acid (2.2 $M^{-1}s^{-1}$ at pH 7.4, 25ºC (Sikora et al., 2009)), and for the H_2O_2-activated uncaging of a mitochondrial uncoupler (10 $M^{-1}s^{-1}$ at pH 8.3, 25ºC) (Quin et al., 2010).

To analyze the reactions of MitoB and MitoP, RP-HPLC was performed at room temperature using a Luna 5 μ Phenyl-Hexyl column (1 x 50 mm, 5 μm) with a Phenyl-Hexyl guard column (2 x 4 mm) (both from Phenomenex) and a Gilson 321 pump. The mobile phase consisted of 0.1% formic acid (FA) in water (buffer A) and 95% acetonitrile (ACN)/0.1% FA (buffer B) delivered as a linear gradient as follows: 0-5 min, 5% B; 5-8 min, 5-25% B; 8-18 min, 25-45% B; 18-26 min, 45-100% B; 26-29 min 100% B; 29-32 min, 100-5% B. The flow rate was 200 μl/min. This analysis showed that the MitoB preparations contained no significant contaminants. After incubation, samples were mixed 1:1 with buffer A and 80 μl was injected into a 50 μl sample loop. The A_{220} of the column eluant was detected using a Gilson UV/Vis 151 spectrophotometer.

To measure the masses of MitoB (both the arylboronic acid and pinacol ester forms), and MitoP, a Waters Quattro Ultima triple quadrupole mass spectrometer was used as is described later. The mobile phase consisted of 50% ACN delivered

continuously into the mass spectrometer at 50 µl/min. To study the reaction of MitoB with H_2O_2, 250 µM MitoB was incubated in KCl medium (120 mM KCl, 10 mM HEPES, 1 mM EGTA; pH 7.0 or 8.0) with 1 mM H_2O_2 at 30ºC, then diluted 1:100 with 95% ACN/0.1% FA and assayed as above.

The production of H_2O_2 by xanthine oxidase (XO) was measured by the horseradish peroxidase oxidation of Amplex Red (Invitrogen) to fluorescent resorufin using a RF-5301 PC fluorimeter (Shimadzu). The excitation and emission wavelengths were 560 and 590 nm, respectively. The concentration of H_2O_2 stock solutions was assessed spectrophotometrically (ε_{240} = 43.6 $M^{-1}cm^{-1}$). XO from microbial sources, catalase from bovine liver and Cu/Zn-SOD from bovine erythrocytes were from Sigma. The slopes for the initial rate of MitoP formation from 100 µM MitoB at pH 8.0, 37ºC upon reaction with H_2O_2 (0-2.5 mM) were determined in duplicate at 285 nm ($\Delta\varepsilon_{MitoP - MitoB}$ = 1,744 $M^{-1}cm^{-1}$), and these were plotted against [H_2O_2] to determine the second order rate constant.

Peroxynitrite ($ONOO^-$) and decomposed $ONOO^-$ were made as described previously (Packer and Murphy, 1994). To assess the effects of $ONOO^-$ on MitoB, 100-250 µM MitoB in KCl medium, pH 8.0 at 37ºC was incubated with $ONOO^-$ (100-250 µM) and compared with the effects of decomposed $ONOO^-$ solutions. The reaction products were analyzed by UV/Vis spectrophotometry and by RP-HPLC. MitoP did not react with 1 mM H_2O_2 (data not shown).

Linoleic acid peroxide was made as described (Ohkawa et al., 1978). For this linoleic acid (Sigma, 0.1 g) was dissolved in 10 ml ethanol and mixed with 100 ml 50 mM sodium borate buffer (pH 9.0) to which was added 2 mg soybean lipoxygenase (BioChemika/Sigma; 7.9 U/mg). The mixture was incubated at 23ºC until the absorbance at 233 nm due to the linoleic acid diene peroxide had reached a stable maximum (~70 min). At this point the mixture was adjusted to pH ~3.0 with 1 M HCl, and the linoleic peroxide was extracted into diethyl ether, dried over sodium sulfate, and the solvent was evaporated under vacuum. The residue was dissolved in ethanol, the concentration of the linoleic acid diene peroxide was determined at 233 nm (ε = 27.4 x 10^3 $M^{-1}cm^{-1}$; Tappel et al., 1952) and a stock solution of ~7 mM was prepared in ethanol.

Mitochondrial Preparation and Experiments

Rat liver and heart mitochondria were prepared by homogenization and differential centrifugation in ice-cold 250 mM sucrose, 10 mM Tris, 1 mM EGTA, pH 7.4, with addition of 0.1% w/v BSA for heart mitochondria. The protein concentration was determined by the biuret assay. Mitochondria were routinely incubated in KCl medium (120 mM KCl, 10 mM HEPES, 1 mM EGTA, pH 7.2) supplemented with 10 mM succinate and 4 µg/ml rotenone. An electrode selective for the TPP moiety of MitoB and MitoP was prepared and used as described (Asin-Cayuela et al., 2004).

The production of H_2O_2 by mitochondria was measured by the horseradish peroxidase-catalyzed oxidation of Amplex Red (Invitrogen) to fluorescent resorufin using a RF-5301 PC fluorimeter (Shimadzu). The excitation and emission wavelengths were 560 and 590 nm, respectively. To do this rat liver mitochondria (1 mg protein/ml) were incubated with 5 µM MitoB, 20 µM Amplex Red, and 4 U/ml horseradish peroxidase ± 200 µM menadione. For some experiments 500 nM FCCP or 100 nM nigericin was present. The assay was calibrated with known amounts of H_2O_2 (ε_{240} = 43.6 $M^{-1}cm^{-1}$).

To extract MitoB and MitoP from mitochondria, samples were centrifuged (2 min at 16,000 x *g*) and the pellet was resuspended in 250 µl of 95% ACN/0.1% FA,

spiked with IS (62.5 pmol d_{15}-MitoP and 62.5 pmol d_{15}-MitoB), vortexed, and centrifuged (10 min at 16,000 x *g*).

Cell Experiments

The toxicity of MitoB and MitoP to C2C12 cells was assessed by incubating cells in 96 well plates with MitoB or MitoP from 0 to 100 μM for 40 h, then the medium was replaced with fresh medium containing 0.05% 3-(4,5-dimethylthiazol-2-yl)-2,5-diphenyltetrazolium bromide (MTT) for 2 h at 37ºC. This was replaced with 20% SDS in 50% dimethylformamide and the absorbance at 570 nm was measured relative to the background absorbance at 650 nm. This MTT assay showed that over 40 h there was negligible toxicity of MitoB or MitoP up to 10 μM, with mild toxicity apparent at 25 μM and above (data not shown).

For MitoB uptake studies, Jurkat cells were incubated at 3 x 10^6 cells/ml in medium supplemented with 5 μM MitoB (Ross et al., 2008). Samples (1 ml) were taken at various time points and cells were pelleted by centrifugation (16,000 x *g* for 2 min). To measure the effect of uncoupling by FCCP on MitoB uptake, Jurkat cells (3 x 10^6/ml) were incubated in PBS supplemented with 1 mM pyruvate ± 500 nM FCCP for 1 h with 5 μM MitoB and then 950 μl of the cell suspension was pelleted by centrifugation (16,000 x *g* for 2 min), the supernatant was discarded, and the pellets snap frozen for LC/MS/MS analysis.

To measure the uptake of [^{3}H]-methylTPP by Jurkat cells, cells were incubated in PBS with 1 mM pyruvate and 500 nM [^{3}H]-methylTPP (100 nCi/ml, American Radiolabelled Chemicals Inc.) at 37ºC. After 1, 2 and 3 h, a 0.5 ml aliquot of the cell suspension was sampled in triplicate and pelleted by centrifugation (16,000 x g for 2 min). The supernatant was removed, and the amount of [^{3}H]-TPMP in the pellet was determined by scintillation counting (Ross et al., 2008). The presence of FCCP decreased the amount of pellet uptake by 47, 53 and 55% at 1, 2 and 3 h respectively.

To extract MitoB and MitoP from adherent C2C12 cells in a 6-well culture dish, the cell medium was removed and the cell layer was incubated with 750 μl 95% ACN/0.1% FA with rocking for 5 min, then scraped and transferred to an

eppendorf tube, and the well washed with 2 x 375 µl 95% ACN/0.1% FA. The combined extracts were spiked with IS (62.5 pmol each of d_{15}-MitoB and d_{15}-MitoP), vortexed, incubated at –20ºC for 30 min and then centrifuged (10 min at 16,000 x *g*).

Fly Culture and Experiments

The *white* Dahomey strain of *D. melanogaster* (negative for the intracellular parasite *Wolbachia*) was used as the wild-type background, which was maintained in a large population cage, allowing outbreeding and overlapping generations, at 25ºC on a 12 h light/dark cycle. All experiments were performed at 25ºC, 65% humidity. Flies were routinely fed a sugar-yeast-agar medium (SYA; 5% (w/v) sugar, 10% (w/v) yeast, 1.5% (w/v) agar, supplemented with the mould inhibitors 3% (v/v) nipagin and 0.3% (v/v) propionic acid, added once the food had cooled down to 60ºC). Eggs were collected onto grape juice agar plates and transferred to SYA bottles at a constant density. Eclosing adults of a defined age were kept as a mixed population for ~48 h to allow mating once, then separated into males and females under CO_2 anaesthesia, and maintained as separate sexes from then on. Flies were aged by transferring adults onto fresh food three times a week. For DR experiments, the level of yeast in the food was varied between 0.5- and 2.0-fold the standard amount (5 and 20% (w/v) respectively). Flies were reared on standard SYA and DR treatment was initiated at 2 d. Paraquat was administered by feeding flies standard SYA supplemented with 20 mM paraquat from a stock in H_2O. For hyperoxia experiments, flies were incubated inside a glove box chamber set at 40 or 65% O_2 using a ProOx controller (BioSpherix). To administer MitoB to flies in their food, standard SYA medium was supplemented with MitoB at a final concentration of 1 mM (from a stock in EtOH at 1% final v/v), added once the food had cooled down to ~50ºC.

To determine the proportion of mitochondria in each fly body segment, the activity of citrate synthase (CS), a mitochondrial matrix enzyme, was measured (Magwere et al., 2006). Homogenates were prepared from whole flies, heads, thoraces and abdomens, using cohorts of 10 flies. Each sample was homogenized on ice in 300 µl of CS assay buffer (100 mM Tris-HCl, 0.1% Triton X-100, pH 8.0), using

three 2-s bursts of a T8 Ultra-Turrax homogenizer (Jencons-PLS), at 1 min intervals. Homogenates were then centrifuged twice at 2000 x *g* for 15 s, the volume of supernatant measured, and the CS activity assayed. To measure CS enzyme activity, samples were added to assay buffer at 25ºC containing 100 mM Tris-HCl, 0.1% Triton X-100, 0.1 mM DTNB and 0.4 mM acetyl-CoA. Change in absorbance at 412 nm was measured for 2 min to determine background activity due to acetyl-CoA hydrolysis, then 0.4 mM oxaloactetate was added to initiate reaction, and absorbance at 412 nm followed for a further 2 min. An extinction coefficient of 13,600 $M^{-1}cm^{-1}$ was used to calculate CS enzyme activity. The protein concentration of each extract was determined in parallel using the bicinchoninic acid assay, and used to calculate the CS specific activity.

Fly mitochondria were isolated (from ~150 7 d females) and incubated as described (Miwa et al., 2003). Mitochondria (50 μg protein) were separated on a 12.5% SDS-PAGE gel and a Western blot was performed using antiserum raised against the TPP moiety (Porteous et al., 2010).

Worm Culture and Experiments

Wild-type *C. elegans* (N2 Bristol strain) was grown and manipulated as published (Sulston and Hodgkin, 1988). Synchronous worm cultures were initiated via alkaline hypochlorite lysis of gravid adults. Eggs were allowed to hatch overnight in M9 buffer (22 mM KH_2PO_4, 42 mM Na_2HPO_4, 86 mM NaCl, 1 mM $MgSO_4$) at 20ºC, and resulting L1 larvae were grown on NGM agar plates seeded with OP50 *E. coli*. At harvest, late L4 larvae and young adult worms were rinsed off the plates with M9 buffer, and washed twice with M9 buffer to remove any bacteria. Aliquots of ~1000 worms were incubated with 10 μM MitoB in M9 buffer at 20ºC under constant agitation (950 rpm) for 1 h in the presence of 50 U catalase, then centrifuged at 16000 x *g* for 2 min at 4ºC. The supernatant was saved, and the worm pellet was washed once in M9 buffer and frozen at –80ºC until LC/MS/MS analysis. For paraquat treatment, worm incubations were supplemented with 0, 50 or 100 mM paraquat.

Direct Infusion of MitoB into Mice

For studies on direct infusion of MitoB, adult male BALB/c mice (~26 g, 12-14 weeks of age) were purchased from Charles River Laboratories (Wilmington, MA). All the studies described were carried out in accordance with the UCL Institutional Animal Care and Use Committee and under UK Home Office license. Instrumentation was performed under isoflurane anesthesia maintained via face mask. A jugular venous line was inserted and tunneled subcutaneously to emerge at the nape of the neck. This was then mounted onto a swivel/tether system secured to the nape using four silk sutures. The animals thus had unrestricted movement within their cage with free access to food and water. After 24 h instrumentation, the mice infusion with saline/5% glucose was initiated at 0.3 ml/h. MitoB was administered in two ways: MitoB was added to the infusate at 300 μM and infused for 2 h from 18 to 20 h after initiation of infusion. From 20 h, the mice were infused with normal saline/5% glucose for a further 4 h; alternatively, MitoB (100 μM in saline/5% glucose) was infused for 6 h from 18 h after initiation of infusion. In both cases 24 h after initiation of infusion the mice were anesthetized with isofluorane and sacrificed. Blood and urine samples were collected by direct cardiac and bladder puncture; heart, liver and kidneys were removed and all were snap frozen on dry ice and stored at –80ºC. In both cases the cumulative dose of MitoB was 180 nmol/mouse, ~7 μmol/kg.

Extraction of MitoB and MitoP from Worms and Mice

To extract MitoB and MitoP from worm pellets, samples were resuspended in 200 μl of 95% ACN/0.1% FA, spiked with IS (100 pmol d_{15}-MitoB and 50 pmol d_{15}-MitoP), vortexed for 5 s, and sonicated in a sonicating waterbath for 10 min. The suspension was centrifuged (10 min at 16,000 x *g*), the supernatant was transferred to a fresh tube, and the pellet was re-extracted with a further 200 μl of 95% ACN/0.1% FA, which was pooled with the first extraction. To extract MitoB and MitoP from mouse tissues, 50 mg tissue was added to a vial and homogenized in 500 μl of 60% ACN/0.1% FA using an Ultraturrax homogeniser, then washed into a fresh eppendorf tube with a further 250 μl of 60% ACN/0.1% FA. At this point the IS (100

pmol d_{15}-MitoB and 50 pmol d_{15}-MitoP) was added and after vortexing the tube was centrifuged (10 min at 16,000 x *g*). The supernatant was transferred to a fresh tube and the pellet was re-extracted with 250 µl of 60% ACN/0.1% FA. The combined supernatants were centrifuged (10 min at 16,000 x *g*) and the supernatant was retained. To extract MitoB and MitoP from urine, samples (~50 µl) were diluted in 500 µl of 60% ACN/0.1% FA, spiked with IS (100 pmol d_{15}-MitoB and 50 pmol d_{15}-MitoB), and centrifuged (10 min at 16,000 x *g*). To extract MitoB and MitoP from blood, samples (50 mg) were homogenized in 500 µl of 95% ACN/0.1% FA, then washed to a fresh eppendorf using a further 250 µl of 95% ACN/0.1% FA, spiked with IS (100 pmol d_{15}-MitoB and 50 pmol d_{15}-MitoB) and incubated on ice for 30 min with occasional vortexing. The homogenate was centrifuged (10 min at 16,000 x *g*), the supernatant was transferred to a fresh tube, and the pellet was re-extracted with 250 µl of 95% ACN/0.1% FA, which was pooled with the first extraction. To assay MitoB and MitoP from cell culture medium or worm incubation medium, samples were spiked with IS (250-500 pmol d_{15}-MitoB and 100 pmol d_{15}-MitoP), and diluted 1:2 with 40% ACN/0.1% FA directly into an autosampler vial.

LC/MS/MS Analysis of MitoB and MitoP

The LC/MS/MS system consisted of a Waters Quattro Ultima triple quadrupole mass spectrometer attached to a binary pump (model 1585; Jasco) and an HTC-PAL autosampler (CTC-Analytics). Samples and standards in autosampler vials were placed in a refrigerated holder (4ºC) while awaiting injection by the autosampler. Liquid chromatography was performed at 30ºC using a Luna 5 µ Phenyl-Hexyl column (1 x 50 mm, 5 µm) with a Phenyl-Hexyl guard column (2 x 4 mm) (both from Phenomenex). The mobile phase consisted of 0.1% FA in water (buffer A) and 95% ACN/0.1% FA (buffer B) delivered as a linear gradient as follows: 0-2 min, 5% B; 2-3 min, 5-25% B; 3-5 min, 25-75% B; 5-7 min, 75-100% B; 7-10 min 100% B; 10-12 min, 100-5% B; 12-20 min, 5% B. The flow rate was 50 µl/min and a 30 µl volume was injected into a 20 µl sample loop. An in-line divert valve was used to divert eluant away from the mass spectrometer from 0-5 min and 16-20 min of the acquisition time. For mass spectrometry, electrospray ionization in positive ion

mode was employed. The instrument parameters were: source spray voltage, 3 kV; cone voltage 100 V; ion source temperature, 80ºC; collision energy, 50 V. Nitrogen was used as the curtain gas and argon as the collision gas. Multiple reaction monitoring (MRM) in positive ion mode was used to detect the compounds. To determine the best fragmentations to use for quantification, direct infusion into the mass spectrometer at 2 µl/min of 1 µM d_{15}-MitoP, MitoP, d_{15}-MitoB pinacol ester or MitoB pinacol ester was used. Published fragmentation patterns for alkylTPP ions (Claereboudt et al., 1993; Denekamp et al., 1999; Denekamp et al., 2003) and the presence of the deuterium atoms solely on the TPP moiety were used to determine the fragmentation (Figure S2). For LC/MS/MS quantification, the following transitions were used: MitoB, 397 > 183; d_{15}-MitoB, 412 > 191; MitoP, 369 > 183 and d_{15}-MitoP, 384 > 191 (see Figure 3A). Data were acquired and analyzed with MassLynx software.

To determine whether there were any products of MitoB other than MitoP, solutions of fly extract were directly infused into the mass spectrometer at 5 µl/min. The daughter ion *m/z* 183 was used to identify parent ions that fragmented to give this product, as this daughter contains the TPP ion and should therefore be present in any MitoB product. Background was determined using solutions extracted from flies with no exposure to MitoB, and MitoB products were determined using solutions extracted from flies that had been incubated with MitoB for 3 h following injection. Data were accumulated over a period of 1 min, and were normalized to total ion count.

SUPPLEMENTAL REFERENCES

Asin-Cayuela, J., Manas, A.R., James, A.M., Smith, R.A.J., and Murphy, M.P. (2004). Fine-tuning the hydrophobicity of a mitochondria-targeted antioxidant. FEBS Lett. *571*, 9-16.

Brand, M.D. (1995). Measurement of mitochondrial protonmotive force. *In* Bioenergetics - a practical approach, G.C. Brown, and C.E. Cooper, eds. (Oxford, IRL), pp. 39-62.

Brand, M.D., Pakay, J.L., Ocloo, A., Kokoszka, J., Wallace, D.C., Brookes, P.S., and Cornwall, E.J. (2005). The basal proton conductance of mitochondria depends on adenine nucleotide translocase content. Biochem J *392*, 353-362.

Claereboudt, J., Baeten, W., Geise, H., and Claeys, M. (1993). Structural characterization of mono- and bisphosphonium salts by fast atom bombardment mass spectrometry and tandem mass spectrometry. Org Mass Spectrometry *28*, 71-82.

Dahm, C.C., Moore, K., and Murphy, M.P. (2006). Persistent S-nitrosation of complex I and other mitochondrial membrane proteins by S-nitrosothiols but not nitric oxide or peroxynitrite: Implications for the interaction of nitric oxide with mitochondria. J Biol Chem. *281*, 10056-10065.

Dawson, M.I., Hobbs, P.D., Kuhlmann, K., Fung, V.A., Helmes, C.T., and Chao, W.R. (1980). Retinoic acid analogues. Synthesis and potential as cancer chemopreventive agents. J Med Chem. *23*, 1013-1022.

Denekamp, C., Pocsfalvi, G., and Claeys, M. (1999). Charge-remote and charge-proximate fragmentations in deuterium-labeled n-hexyadecyltriphenylphosphonium cations. Int J Mass Spect *188*, 163-175.

Denekamp, C., Tenetov, E., and Horev, Y. (2003). Homolytic cleavages in pyridinium ions, an excited state process. J Amer Soc Mass Spectrometry *14*, 790-801.

Dickinson, B.C., and Chang, C.J. (2008). A targetable fluorescent probe for imaging hydrogen peroxide in the mitochondria of living cells. J Amer Chem Soc *130*, 9638-9639.

Folk, D.G., Han, C., and Bradley, T.J. (2001). Water acquisition and partitioning in Drosophila melanogaster: effects of selection for desiccation-resistance. J Exp Biol *204*, 3323-3331.

Howard, O.M., Dong, H.F., Oppenheim, J.J., Insaf, S., Santhosh, K.C., Paul, G., and Cushman, M. (2001). Inhibition of RANTES/CCR1-mediated chemotaxis by cosalane and related compounds. Bioorg Med Chem Lett *11*, 59-62.

Ishiyama, T., Takagi, J., Ishida, K., Miyaura, N., Anastasi, N.R., and Hartwig, J.F. (2002). Mild iridium-catalyzed borylation of arenes. High turnover numbers, room temperature reactions, and isolation of a potential intermediate. J Amer Chem Soc *124*, 390-391.

Kelso, G.F., Porteous, C.M., Coulter, C.V., Hughes, G., Porteous, W.K., Ledgerwood, E.C., Smith, R.A.J., and Murphy, M.P. (2001). Selective targeting of a redox-active ubiquinone to mitochondria within cells. J Biol Chem ***276***, 4588-4596.

Kwong, L. K., Mockett, R. J., Bayne, A.-C. V., Orr, W. C., and Sohal, R. (2000). Decreased mitochondrial hydrogen peroxide release in transgenic *Drosophila melanogaster* expressing intramitochondrial catalase. Arch Biochem Biophys *383*, 303-308

Magwere, T., Goodall, S., Skepper, J., Mair, W., Brand, M.D., and Partridge, L. (2006). The effect of dietary restriction on mitochondrial protein density and flight muscle mitochondrial morphology in *Drosophila*. J Gerontol A Biol Sci Med Sci *61*, 36-47.

Miller, E.W., Tulyathan, O., Isacoff, E.Y., and Chang, C.J. (2007). Molecular imaging of hydrogen peroxide produced for cell signaling. Nat Chem Biol *3*, 263-267.

Miwa S., St-Pierre J., Partridge L., and Brand M.D. (2003). Superoxide and hydrogen peroxide production by *Drosophila* mitochondria. Free Rad Biol Med *35*, 938-948.

Ohkawa, H., Ohishi, N., and Yagi, K. (1978). Reaction of linoleic acid hydroperoxide with thiobarbituric acid. J Lipid Res *19*, 1053-1057.

Packer, M.A., and Murphy, M.P. (1994). Peroxynitrite causes calcium efflux from mitochondria which is prevented by Cyclosporin A. FEBS Letts *345*, 237-240.

Porteous, C.M. Logan, A., Evans, C., Ledgerwood, E.C., Menon, D. K., Aigbirhio, F., Smith, R.A.J., and Murphy, M.P. (2010). Rapid uptake of lipophilic triphenylphosphonium cations by mitochondria *in vivo* following intravenous injection: implications for mitochondria-specific therapies and probes. Biophys. Biochim. Acta. *1800*, 1009-1017.

Quin, C., Robertson, L., McQuaker, S.J., Price, N.C., Brand, M.D., and Hartley, R.C. (2010). Caged mitochondrial uncouplers that are released in response to hydrogen peroxide. Tetrahedron *66*, 2384-2389.

Ross, M.F., Prime, T.A., Abakumova, I., James, A.M., Porteous, C.M., Smith, R.A.J., and Murphy, M.P. (2008). Rapid and extensive uptake and activation of hydrophobic triphenylphosphonium cations within cells. Biochem J *411*, 633-645.

Sikora, A., Zielonka, J., Lopez, M., Joseph, J., and Kalyanaraman, B. (2009). Direct oxidation of boronates by peroxynitrite: mechanism and implications in fluorescence imaging of peroxynitrite. Free Radic Biol Med *47*, 1401-1407.

Smith, R.A.J., Porteous, C.M., Gane, A.M., and Murphy, M.P. (2003). Delivery of bioactive molecules to mitochondria in vivo. Proc Natl Acad Sci USA *100*, 5407-5412.

Sulston, J., and Hodgkin (1988). Methods. In The nematode C elegans W.B. Wood, ed. (New York, Cold Spring Harbor Laboratory Press), pp. 587-606.

Tappel, A. L., Boyer, P. D., and Lundberg, W. O. (1952). The reaction mechanism of soy bean lipoxidase. J Biol Chem. *199* 267-281.

Xu, J., Duran, D., and Mao, B. (2006). On-column hydrolysis kinetics of boronic pinacol ester intermediates for use in optimization of fast HPLC methods. J Liq Chrom & Related Technologies *29*, 2795-2806.
